# Surface Stability and Growth Kinetics of Compound Semiconductors: An *Ab Initio*-Based Approach

**DOI:** 10.3390/ma6083309

**Published:** 2013-08-06

**Authors:** Yoshihiro Kangawa, Toru Akiyama, Tomonori Ito, Kenji Shiraishi, Takashi Nakayama

**Affiliations:** 1 Research Institute for Applied Mechanics, Kyushu University, 6-1 Kasuga-koen, Kasuga, Fukuoka 816-8580, Japan; 2 Department of Physics Engineering, Mie University, 1577 Kurima-Machiya, Tsu 514-8507, Japan; E-Mails: akiyama@phen.mie-u.ac.jp (T.A.); tom@phen.mie-u.ac.jp (T.I.); 3 Institute of Physics, University of Tsukuba, 1-1-1 Tennodai, Tsukuba, Ibaraki 305-8577, Japan; E-Mail: shiraishi@comas.frsc.tsukuba.ac.jp; 4 Department of Physics, Faculty of Science, Chiba University, 1-33 Yayoi, Inage, Chiba 263-8522, Japan; E-Mail: nakayama@physics.s.chiba-u.ac.jp

**Keywords:** *ab initio* calculation, compound semiconductor, surface phase diagram

## Abstract

We review the surface stability and growth kinetics of III-V and III-nitride semiconductors. The theoretical approach used in these studies is based on *ab initio* calculations and includes gas-phase free energy. With this method, we can investigate the influence of growth conditions, such as partial pressure and temperature, on the surface stability and growth kinetics. First, we examine the feasibility of this approach by comparing calculated surface phase diagrams of GaAs(001) with experimental results. In addition, the Ga diffusion length on GaAs(001) during molecular beam epitaxy is discussed. Next, this approach is systematically applied to the reconstruction, adsorption and incorporation on various nitride semiconductor surfaces. The calculated results for nitride semiconductor surface reconstructions with polar, nonpolar, and semipolar orientations suggest that adlayer reconstructions generally appear on the polar and the semipolar surfaces. However, the stable ideal surface without adsorption is found on the nonpolar surfaces because the ideal surface satisfies the electron counting rule. Finally, the stability of hydrogen and the incorporation mechanisms of Mg and C during metalorganic vapor phase epitaxy are discussed.

## 1. Introduction

### 1.1. Scope of Review

Current semiconductor devices, such as optical and electronic devices, are fabricated using the vapor phase epitaxy (VPE) technique whereby a gas-solid interface is formed at the growth front. It is important to control the interface mass transfer when fabricating compositionally controlled semiconductor thin films. It is well known that reconstructed structures appear on the growth front (surfaces) of semiconductor materials [1]. Therefore, we need to understand the atomic structures on the surfaces to control the interface mass transfer. To date, many theoretical works have investigated the surface structures of semiconductors [2,3,4]. Kaxiras *et al.* [2] studied the lowest-energy geometry for GaAs(111) using different stoichiometries. Qian *et al.* [3] discussed the relationship between the stoichiometry and the surface reconstruction on GaAs(001) using chemical potentials. Northrup [4] classified the stable structures on Si(001)H using the chemical potential of H. However, all of these approaches discussed the static structural surface stability at 0 K, even though their methodologies were different. Generally, VPE, such as molecular beam epitaxy (MBE) and metalorganic vapor phase epitaxy (MOVPE), is performed under finite temperatures and gas pressures. Therefore, it is necessary to consider the ambient conditions when predicting the reconstructed structures on the growth surfaces. In 2001, we [5,6] proposed an *ab initio*-based approach that incorporates the gas-phase free energy. The theoretical approach is useful for analyzing the influence of temperature and pressure on the stability of the reconstructed surfaces. By applying this method, growth kinetics and processes can be investigated. The theoretical approach has been modified for studying reconstructions on various semiconductor surfaces. In 2002, Van de Walle and Neugebauer [7] reported the surface phase diagram for hydrogen on GaN surfaces. Shu *et al.* [8] revealed the thermodynamic phase diagram for hydrogen on InP(111)B. In this review article, we discuss reconstructed structure stabilities and elementary growth processes on GaAs and III-nitride surfaces during MBE and MBE/MOVPE processes, respectively.

### 1.2. Prior Works in the Field of GaAs MBE

Electronic devices, such as GaAs field effect transistors (FETs), have been fabricated using MBE since the 1970s [9]. In the 1970s, researchers reported that relatively smooth surfaces could be obtained using MBE and that rough surfaces could be formed using chemical vapor deposition (CVD). In the 1980s, atomically flat surfaces could be obtained easily by MBE [10,11], which was confirmed from the specular beam intensity oscillation in the reflection high energy electron diffraction (RHEED) pattern during MBE. RHEED analyses can also be used to investigate the two-dimensional periodicities of reconstructed structures grown on surfaces [12]. Later, GaAs surface phase diagrams were reported as a function of the temperature and the beam equivalent pressure (BEP) ratio, BEP(As_4_)/BEP(Ga), during MBE [1]. In addition, many studies have investigated the growth kinetics and processes of GaAs MBE. For example, Shiraishi and Ito [13,14] performed theoretical investigations concerning the adsorption and migration behavior of Ga on GaAs. Ito *et al.* [15] used Monte Carlo (MC) simulations and scanning tunneling microscopy (STM) analyses to reveal that an island growth process occurs on reconstructed GaAs surfaces. Nishinaga *et al.* [16] used a microprobe-RHEED and scanning electron microscopy (SEM) installed MBE to examine the surface diffusion of Ga on vicinal GaAs surfaces. As described above, research in the field of GaAs MBE has a long history, and there is extensive knowledge of the surface stability and growth kinetics. In the present review article, we confirm the feasibility of our *ab initio*-based approach by comparing calculated results with experiments.

### 1.3. Issues with III-Nitride MOVPE

Because of the successful fabrication of high-quality epitaxial GaN crystals [17,18] and the development of GaN-based optoelectronic devices in the 1990s [19], nitride semiconductors, such as AlN, GaN, and InN, have emerged as very important material systems. These semiconductors have a unique suitability for light emission over a wide range of wavelengths that was previously not accessible with solid-state light emitters. To improve the device performance of these materials, strict control over the growth conditions and a thorough understanding of surface reconstructions is essential. Indeed, the surface structure determines the morphology, the host-atom, impurity incorporation, and, ultimately, the crystal quality. Therefore, the surface reconstruction and growth kinetics of nitride semiconductor surfaces are important at various stages in current technological processes, and an understanding of the physics and chemistry is of great interest.

In the field of bright green-light emitting diode (LED) development, III-nitride growth on nonpolar and semipolar surfaces is attracting increasing attention. There are large piezoelectric fields in the III-nitrides that have a wurtzite structure. In addition, the active regions of typical InGaN LEDs are under biaxial compressive stress due to the larger lattice constant of InGaN compared with a GaN substrate. Consequently, InGaN quantum wells (QWs) grown along the [0001] (*c*-axis) direction exhibit an internal piezoelectric field, and electrons and holes are separated to opposite interfaces of the QW. This spatial separation of electrons and holes in the QW affects the quantum efficiency of LEDs. To overcome this problem, III-nitrides should be grown along the crystallographic directions where the piezoelectric fields are negligible. Takeuchi *et al.* [20] theoretically predicted that III-nitride growth on nonpolar and semipolar surfaces is essential to reduce the piezoelectric fields in QWs. In the present review article, we discuss the surface stability of AlN, GaN and InN with various orientations, such as polar, nonpolar and semipolar surfaces. The role of H adsorption in surface stability and the roles of Mg and C incorporation during growth are also discussed.

## 2. Methodology

Two types of processes contribute to understanding the stability of surface structures under growth conditions. First, we investigate the relative stability among various surface structures. To determine the relative stability, a conventional approach [2,3,4] is useful, which is based on the surface formation energy and chemical potentials. In Section 2.1.2, we introduce the approach for III-nitride surfaces. Next, constituent atom adsorption-desorption behaviors on the stable surfaces that are revealed by the conventional approach are studied to create surface phase diagrams as a function of temperature and vapor pressure. In Section 2.1 and Section 2.1.1, we describe how to construct a surface phase diagram using our *ab initio*-based approach, which takes into account the gas-phase free energy.

### 2.1. *Ab Initio*-based Approach Incorporating Gas-phase Free Energy

**Figure 1:**
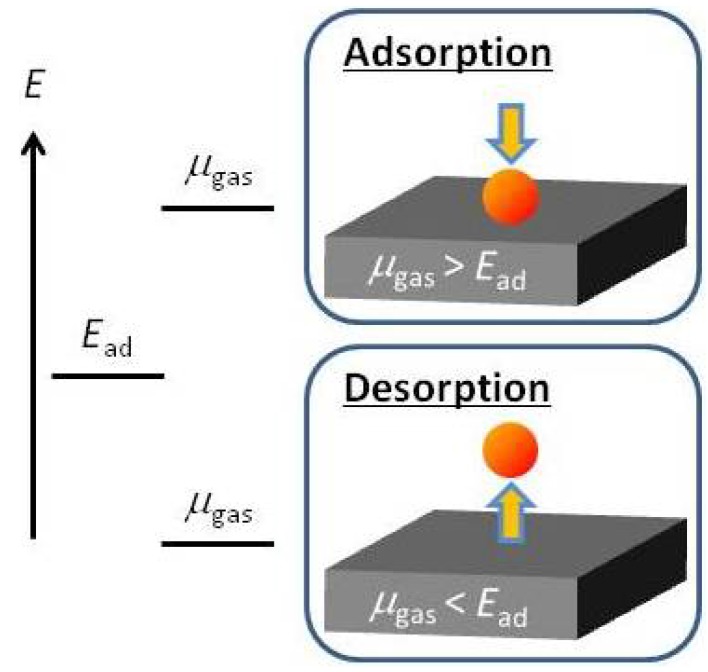
Schematic of the *ab initio*-based approach. By comparing the values of the chemical potential, *μ*_gas_, with adsorption energy, *E*_ad_, we can discuss the adsorption-desorption behavior of an adatom (a molecule).

To control the interface mass transfer, it is necessary to understand the adsorption-desorption behavior of adatoms (molecules) on the surfaces and to understand the structural stability of the growth surfaces. Thus, we proposed an *ab initio*-based approach that incorporates the free energy of the gas phase [5,6]. The concept of this theoretical approach is presented in Figure 1. As you can see, an impinging atom (molecule) can adsorb on the surface if the free energy of the atom (molecule) in the gas phase is larger than its adsorption energy. In contrast, an impinging atom (molecule) desorbs if its gas-phase free energy is smaller than the adsorption energy. The free energy or chemical potential (*μ*_gas_) of an atom (molecule) can be computed using quantum statistical mechanics. The adsorption energy (*E*_ad_) can be obtained using *ab initio* calculations. The adsorption energy considered in this study is the energy difference between the two slab models. One model is a surface with an adatom, and the other is a surface without an adatom, *i.e.*, the adatom is in the vacuum region. The adsorption-desorption activation energy is not considered because we consider the statics instead of the kinetics to construct the surface phase diagrams. However, the activation energy should be considered if the growth kinetics are investigated. By comparing *μ*_gas_ with *E*_ad_, we can discuss the adsorption-desorption behavior, as presented in Figure 1. The Gibbs free energy of formation vibrational contribution is very small compared with the energy difference between a given structure and the ideal surface [7,8]. Thus, when the temperature or pressure is varied, the gas-phase entropy difference is also considerably larger than the surface entropy change. Therefore, only the entropic effects of the gas phase are considered throughout our theoretical approach.

The chemical potential, *μ*_gas_, for the ideal gas is given by [21]:

(1) $$\mu_{\text{gas}}=-k_{\text{B}}T\text{ ln}(gk_{\text{B}}T/p\;\times \varsigma_{trans}\varsigma_{rot}\varsigma_{vibr})$$

(2) $$\varsigma_{\text{trans}}={\left(2\pi mk_{\text{B}}T/h^{2}\right)}^{3/2}$$

(3) $$\varsigma_{\text{rot}}=(1/\pi \sigma ){\left\{8\pi^{3}{\left({\text{I}}_{\text{A}}{\text{I}}_{\text{B}}\cdots \right)}^{1/n}k_{\text{B}}T/h^{2}\right\}}^{n/2}$$

(4) $$\varsigma_{\text{vibr}}={\text{Π}}_{\text{i}}^{3N-3-n}{\left\{1-\text{exp}(-h\nu_{\text{i}}/k_{\text{B}}T)\right\}}^{-1}$$

where ζ_trans_, ζ_rot_ and ζ_vibr_ are the partition functions for the translational motion, the rotational motion and the vibrational motion, respectively. Here, *k*_B_ is Boltzmann’s constant, *T* is the temperature, *g* is the degree of degeneracy of the electron energy level (see Table 1), *p* is the BEP of the particle, *m* is the mass of one particle, *h* is Planck’s constant, σ is the symmetric factor, *I*_I_ is the moment of inertia, *n* is the degree of freedom of the rotation, *N* is the number of atoms in the particle, *i* is the degree of freedom for the vibration, and ν is the frequency. *I*_I_ is written as

(5) $$I_{\text{I}}=m_{\text{I}}r^{2}$$

where *m*_I_ is the reduced mass, and *r* is the radius of gyration.

**Table 1:** Electron energy level degeneracy of some elements.

Group	Element	*g*
I	H, Li, Na, K, Rb, Cs, Cu, Ag, Au	2
II	Be, Mg, Ca, Sr, Ba, Zn, Cd, Hg	1
III	B, Al, Ga, In, Tl	2
IV	C, Si, Ge, Sn, Pb	3
V	N, P, As, Sb, Bi	4
VI	O, S, Se, Te, Po	3
VII	F, Cl, Br, I	2
0	He, Ne, Ar, Kr, Xe, Rn	1

The adsorption energies of adatoms (molecules) were obtained by *ab initio* calculations. Details are written below.

#### 2.1.1. Computational Approach for GaAs Surfaces

For the total-energy calculations of the GaAs systems, we used the *ab initio* pseudopotential method based on the local-density functional formalism [22]. We adopted the Kleinman-Bylanders separable pseudopotentials method, and the local potential cut-off value was carefully chosen to prevent ghost bands [23]. The conventional repeated slab geometry was employed to simulate the surface. The unit super lattice consists of fictitious H atoms and a vacuum region equivalent to a thickness of approximately 15 atomic layers. The thickness validity in the repeated slab model was carefully examined.

To investigate the stability of an As_2_ or As-dimer on the GaAs(001)-c(4 × 4), we computed *μ*_As2_ in the gas phase. As can be seen in Equations (1)–(5), ν and *r* are the unknown parameters needed for computing *μ*_As2_. In this study, *ab initio* molecular orbital calculations were performed to estimate these parameters using the Gaussian 98 program [24]. The parameters of ν and *r* for As_2_ and other molecules that appeared upon MBE of typical semiconductors are listed in Table 2. In the calculations, Becke’s hybrid Hartree-Fock density functional method (B3LYP) [25] was used. The second-order Møller-Plesset perturbation calculations (MP2) were also performed for comparison. The basis sets used in the calculations were Huzinaga’s MIDI-4** [26] for the element on and after the 3rd period in the periodic table and were Pople’s 6-31G** for the element on and before the 3rd period. As presented in Table 2, we found that the calculated values of ν for H_2_ and N_2_ at the B3LYP level are close to the experimental values [27], in contrast to those obtained at the MP2 level. This finding suggests that the calculations at the B3LYP level are more suitable for the prediction of ν than the calculations at the MP2 level. Therefore, we used the parameters of ν and *r* obtained at the B3LYP/MIDI-4** level for the calculations of the As_2_ chemical potential, *μ*_As2_, in the present study.

**Table 2:** Diatomic molecule (cm^−1^) frequencies. The values in parentheses are the optimized bond lengths (Å). B3LYP and MP2 represent the calculations at the B3LYP/Y and MP2/Y (Y = MIDI-4** or 6-31G**) levels, respectively. The experimental values are those obtained by Huber *et al.* [27].

Molecule	B3LYP		MP2		Exp.
	MIDI-4**	6-31G**	MIDI-4**	6-31G**	
H_2_	–	4467 (0.7427)	–	4609 (0.7338)	4401
N_2_	–	2458 (1.1055)	–	2180 (1.1300)	2360
P_2_	792 (1.9217)	796 (1.9044)	696 (1.9648)	717 (1.9323)	–
As_2_	446 (2.1242)	–	385 (2.1694)	–	–

#### 2.1.2. Computational Approach for III-nitride Surfaces

The III-nitride total-energy calculations were performed using the plane-wave pseudopotential approach and the generalized gradient approximation [28]. We used the norm-conserving pseudopotentials [29] for Ga and H atoms and the ultrasoft pseudopotential [30] for N atoms. Ga 3d electrons were treated by partial core corrections [31]. The conjugate-gradient technique was utilized for both the electronic structure calculations and for geometry optimization [32,33]. The geometry optimization was performed until the remaining forces acting on the atoms were less than 5.0 × 10^−3^ Ry/Å. The valence wave functions were expanded by the plane-wave basis set with a cut-off energy of 28 Ry. Details of the calculation models are written elsewhere [34,35,36,37,38,39,40,41,42,43,44,45,46,47,48,49,50].

The relative stability among various surfaces (in the case of GaN) was assessed using the surface formation energy, *E_f_*. This was estimated using the following equation [7,51,52]:

(6) $$E_{f}=E_{tot}-E_{ref}-\sum_{i}n_{i}\mu_{i}$$

where *E*_tot_ and *E*_ref _are the total energy of the surface under consideration and the total energy of the reference surface, respectively; *μ_i_* is the chemical potential of the *i*th species; and *n_i_* is the number of excess or deficit *i*th atoms with respect to the reference. Here, the surface is assumed to be in equilibrium with the bulk GaN, as expressed by

(7) $$\mu_{Ga}+\mu_{N}=\mu_{GaN}^{bulk}$$

where $\mu_{GaN}^{bulk}$ is the chemical potential of bulk GaN. The *μ*_Ga_ can vary in the thermodynamically allowed range of $\mu_{Ga}^{bulk}+\Delta H_{f}\leq \mu_{Ga}\leq \mu_{Ga}^{bulk}$, where $\Delta H_{f}$ is the heat of formation of bulk GaN and $\mu_{Ga}^{bulk}$ is the chemical potential of bulk Ga. The lower and upper limits correspond to N-rich and Ga-rich conditions, respectively. The same formalism can be applied to the study of AlN and InN surfaces using the chemical potentials of bulk Al and AlN ($\mu_{Al}^{bulk}$ and $\mu_{AlN}^{bulk}$) and bulk In and InN ($\mu_{In}^{bulk}$ and $\mu_{InN}^{bulk}$) as a function of Al and In chemical potentials, $\mu_{Al}$ and $\mu_{In}$, respectively. The calculated values of $\Delta H_{f}$ are −2.78 eV for AlN [48,49], −1.24 eV for GaN [42,43,44,45], and −0.37 eV for InN [38,39,41].

### 2.2. Monte Carlo Simulation

To investigate the adatom diffusion length while it is on the surface, we performed Monte Carlo (MC) random-walk simulations [53]. In the simulation procedure, specific lattice sites for an adatom on the surface are assumed, *i.e.*, a discrete lattice-gas model is employed. The site-correlated adsorption probability *P*_ad_(*x*) is written, assuming the local-thermal equilibrium approximation, by

(8) $$P_{\text{ad}}(x)=\text{exp}(-\Delta \mu (x)/k_{\text{B}}T)/\{1+\text{exp}(-\Delta \mu (x)/k_{\text{B}}T)\}$$

where Δ*μ*(*x*) is the difference in chemical potential between when an atom is on the site *x* (*μ*_ad_(*x*)) and when it is in the gas phase (*μ*_gas_). That is, Δ*μ*(*x*) = *μ*_ad_(*x*) − *μ*_gas_. Here, the chemical potential of an atom on the surface *μ*_ad_(*x*) corresponds to a negative desorption energy *E*_de_(*x*). The chemical potential of the atom in the gas phase *μ*_gas_ is given by Equation (1). The diffusion probability *P*_diff_(*x*→*x*′) is assumed to be in the Arrhenius form of

(9) $$P_{\text{diff}}(x\to x^{\prime })=\nu_{\text{lattice}}\exp\left\{-\Delta E(x\to x^{\prime })/k_{\text{B}}T\right\}$$

where the diffusion pre-factor ν_lattice_ is 2*k*_B_*T*/*h* [54], and Δ*E*(*x*→*x*′) is the local activation energy for the adatom hopping from site *x* to *x*′. The desorption probability *P*_de_(*x*) is written by

(10) $$P_{\text{de}}(x)=\nu_{\text{lattice}}\text{exp}[-\{(E_{\text{de}}\left(x\right)-\Delta \mu \left(x\right))/k_{\text{B}}T\}]$$

This equation suggests that the chemical potential difference, Δ*μ*(*x*) [=*μ*_ad_(*x*) − *μ*_gas_], between the atom on the surface and the atom in the gas phase influences the desorption activation energy of the atom. Thus, the adatom easily desorbs if *μ*_gas_ is lower than *μ*_ad_(*x*), but the atom prefers to stay on the surface if *μ*_gas_ is higher than *μ*_ad_(*x*). More precisely, the probability of overcoming the activation energy of *E*_de_(*x*) {=exp[−*E*_de_(*x*)/*k*_B_*T*]} is reduced (or enhanced) by a weighting function of exp[Δ*μ*(*x*)/*k*_B_*T*], which corresponds to the local-thermal equilibrium desorption probability. On the basis of the above-mentioned stochastic differential equation, we performed the MC random-walk simulations.

## 3. Applications to GaAs Surfaces

In this section, we discuss the feasibility of the *ab initio*-based approach that incorporates the free energy of the gas phase. In the conventional GaAs MBE system, Ga atoms and As_2_ molecules are supplied on the growth surface at a certain finite temperature. Depending on the growth conditions, *i.e.*, BEP and temperature, some stable reconstructed structures appear on the growth surfaces. For example, GaAs(001)-(4 × 2)β2 is observed when Ga-rich conditions are used, and GaAs(001)-c(4 × 4) can be observed when As-rich conditions are used. Here, we analyzed the stable conditions of these reconstructed structures, *i.e.*, the surface phase diagrams of GaAs(001), and compared them with experimental results.

### 3.1. Surface Phase Diagram

#### 3.1.1. GaAs(001)-(4 × 2)β2

**Figure 2:**
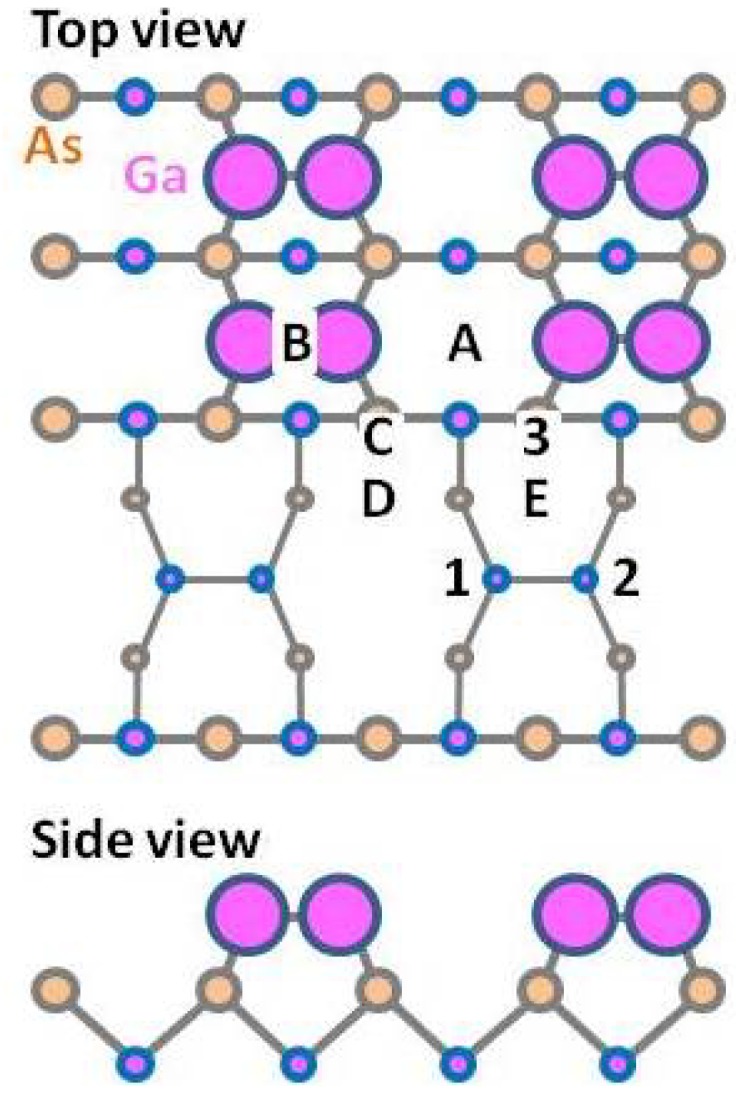
Schematic of the GaAs(001)-(4 × 2)β2 surface. Adsorption sites are indicated by the letters A–E.

**Figure 3:**
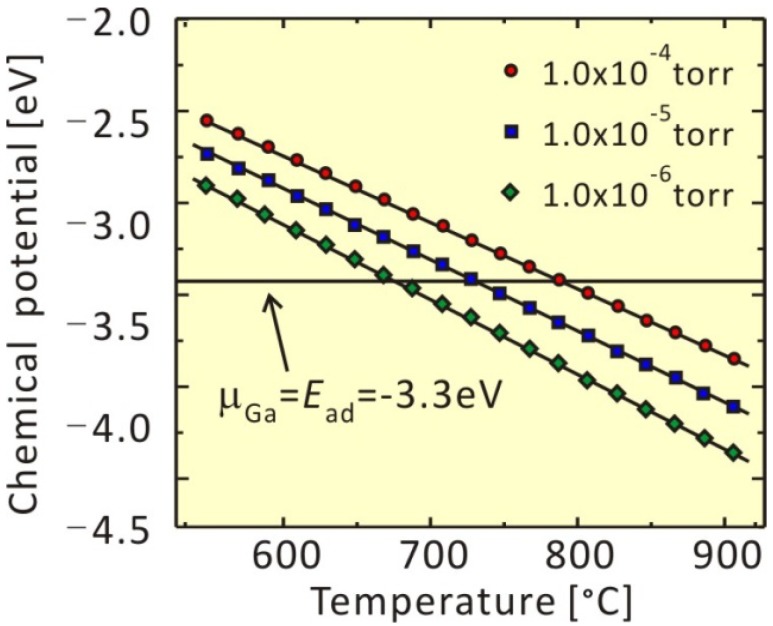
Chemical potential, *μ*_gas_, as a function of temperature.

**Figure 4:**
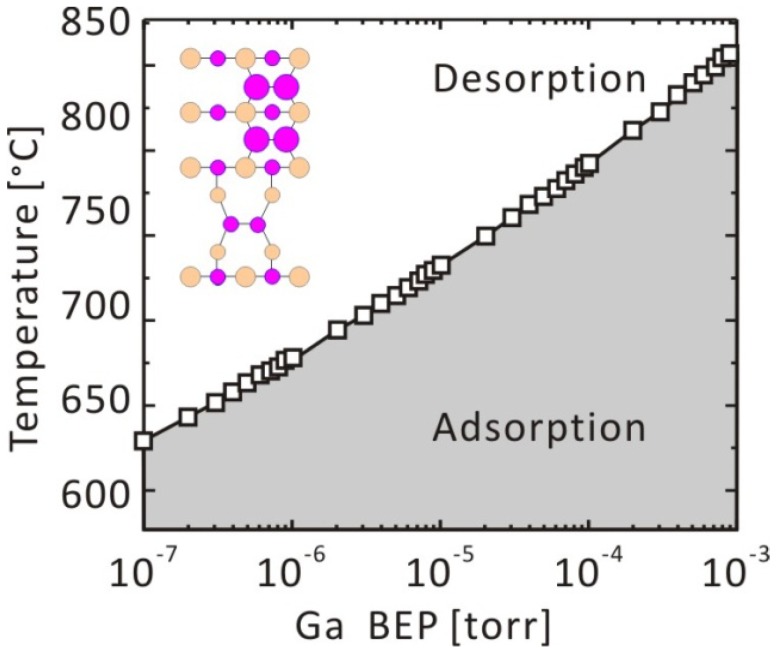
Pressure-temperature phase diagram for the GaAs(001)-(4 × 2)β2 surface.

The atomic arrangement of GaAs(001)-(4 × 2)β2 is presented in Figure 2. We investigated the adsorption-desorption behavior of Ga on this surface to discuss the boundary between the -(4 × 2)β2 stable (desorption preferable) conditions and Ga-droplet formation (adsorption preferable) conditions. This structural model was used to demonstrate the feasibility of the theoretical approach. Recently, the GaAs(001)-(4 × 2)ζ surface model was proposed [55,56]. The calculated results suggest that the Ga adatom prefers the “E” surface site, where two-Ga–Ga bonds (E-1 and E-2) and a Ga–As bond (E-3) are formed around the adatom. The adsorption energies, E_ad_, can be calculated as the difference between the total energy when the Ga adatom is at the E-site and when the Ga is in the vacuum region. The Ga adsorption energy at the E-site is estimated to be ~−3.3 eV. This suggests that the Ga droplet would be formed when *μ*_Ga_ > −3.3 eV; however, -(4 × 2)β2 is stable when *μ*_Ga_ < −3.3 eV. Figure 3 shows the *μ*_Ga_ as a function of temperature. Here, the *μ*_Ga_ line, which is under the condition of Ga-BEP (*p*_Ga_) at 1.0 × 10^−5^ Torr, crosses the line of *μ*_Ga_ = *E*_ad_ = −3.3 eV at approximately 1000 K. This suggests that the critical temperature for Ga adsorption is ~1000 K at a *p*_Ga_ = 1.0 × 10^−5^ Torr. The critical temperatures for Ga adsorption under the various BEP conditions are plotted on the *p*-*T* diagram presented in Figure 4. This surface phase diagram shows that the Ga-droplet would be appear at low temperature and in the high Ga-BEP region, but -(4 × 2)β2 is stable at a high temperature and in the low Ga-BEP region, which is a reasonable result. Furthermore, the following phenomena are reported: (1) Ga-droplets are observed under ~900 K during the GaAs MBE under Ga-rich conditions [57]; and (2) Ga desorption proceeds above ~970 K after turning off the Ga flux [58,59]. The experimental results agree with our calculated results. Thus, the feasibility of our *ab initio*-based approach that incorporates the free energy of the gas phase is confirmed.

#### 3.1.2. GaAs(001)-c(4 × 4)

**Figure 5:**
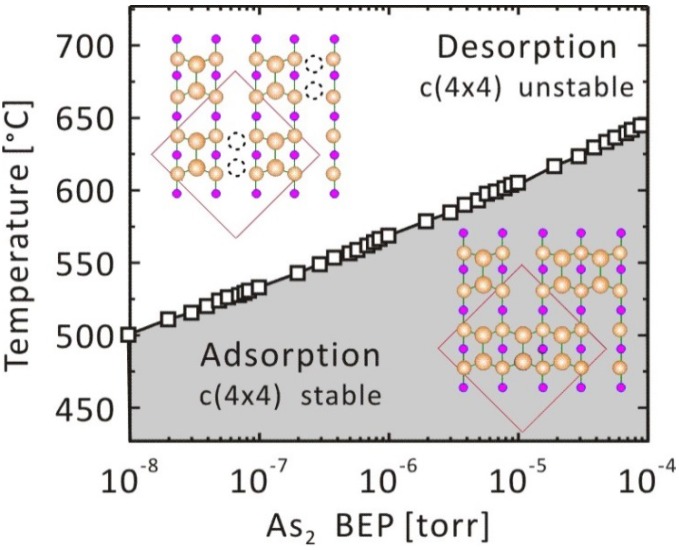
Pressure-temperature phase diagram for the GaAs(001)-c(4 × 4).

The stability of the As-dimer on the GaAs(001)-c(4 × 4) As-rich surface was studied. The -c(4 × 4) stable conditions were estimated by comparing the adsorption energy when the As-dimer forms -c(4 × 4), *E*_ad-As2_ (= −3.6 eV/dimer [60]), with *μ*_As2_. The desorption of the As-dimer from the topmost layer proceeds, and the -c(4 × 4) surface would be unstable when *μ*_As2_ < *E*_ad-As2_, whereas -c(4 × 4) becomes stable when *μ*_As2_ > *E*_ad-As2_. Figure 5 shows the *p*-*T* phase diagram for GaAs(001)-c(4 × 4). In Figure 5, we find that the -c(4 × 4) reconstructed structure is stable at higher BEP and in the lower temperature region. This agrees with experimental results, *i.e.*, the -c(4 × 4)-like region appears at *T* < 773 K under the condition of *p*_As2_ = 6 × 10^−7^ Torr [61], while the -c(4 × 4)-like region is observed at *T* = 853 K under the condition of *p*_As2_ = 8 × 10^−7^ Torr [15]. The result suggests that our computational method is feasible to predict the adsorption–desorption behavior of the As_2_ molecule. Other investigations have been conducted to determine the boundaries among transition states composed of Ga–As and Ga–Ga dimers [62,63].

### 3.2. Growth Kinetics

**Figure 6:**
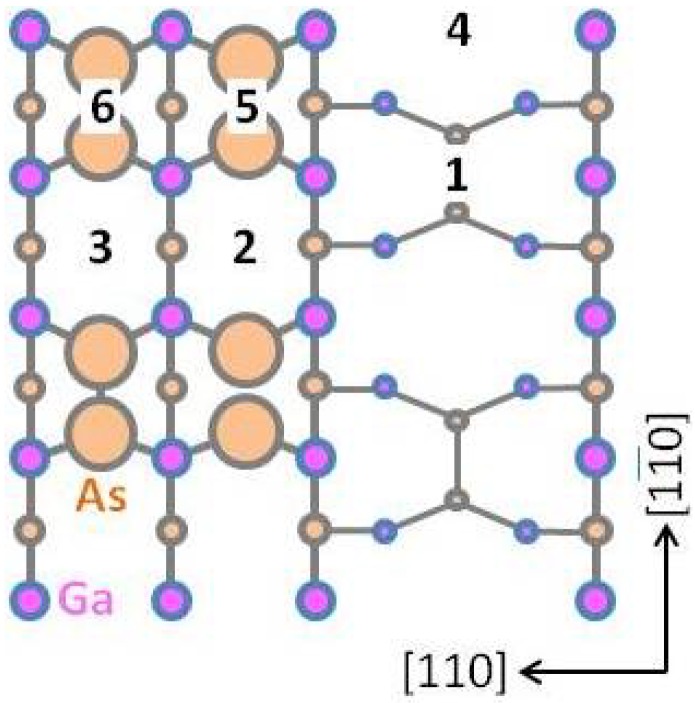
Plane view of GaAs(001)-(2 × 4)β2. Adsorption sites for Ga are indicated by numbers. The migration barriers and desorption energies are presented in Table 3.

**Figure 7:**
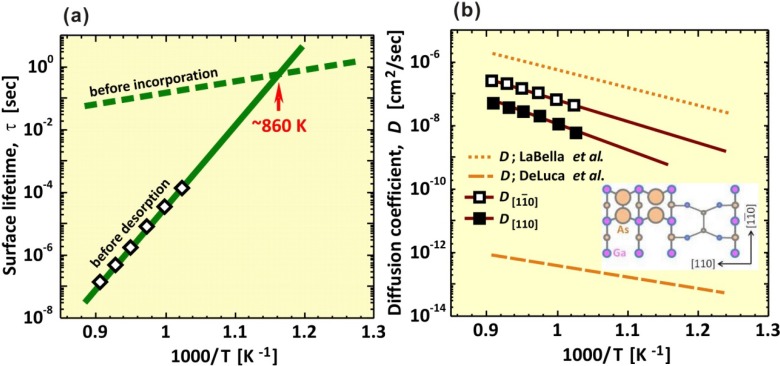
(**a**) Ga surface lifetime, *τ*; and (**b**) diffusion coefficient, *D*, as a function of reciprocal temperature. Green solid and dashed lines are the calculated *τ* before desorption and *τ* before incorporation [66], respectively. Brown solid lines with open and filled squares are the $D_{\left[1\bar{1}0\right]}$ and *D*_[110]_, respectively. The experimental results for the Ga diffusion coefficient are also presented in the diagram by orange dotted (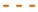) [69] and dashed lines (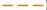) [65].

The Ga diffusion length on the well-ordered GaAs(001)-(2 × 4) β2 surface was studied. Figure 6 presents a schematic of the -(2 × 4)β2 surface. The migration barriers from *x* to *x*′ site and the Ga desorption energy from the *x* site on the -(2 × 4)β2 surface are shown in Table 3. In the MC random-walk simulation, the two-dimensional periodic boundary conditions were employed to the potential surface, *i.e.*, an extremely flat and defect-free surface was considered. Recently, a more precise Ga migration potential on GaAs(001)-(2 × 4)β2 was reported [64]. If their data matrix is applied to the MC simulation, more precise properties could be obtained. In the present MC simulations, we applied the coarse data matrix presented in Figure 6 to confirm the feasibility of the simple model described in Section 2.2. First, we compared the calculated surface lifetime, *τ*, and diffusion coefficient, *D*, with those obtained from experiments. Then, we discuss the diffusion length, *L*, of Ga on the -(2 × 4)β2 surface because *L* is generally given by $L=\sqrt{2D\tau }$. Figure 7a,b shows the Ga *τ* and *D*, respectively, as a function of the reciprocal temperature. The green solid and dashed lines in Figure 7a represent the calculated Ga surface lifetime before desorption and before incorporation, respectively, as estimated by the ion-beam technique [65]. The experiments were conducted using GaAs(001) that was misoriented by 2.3° ± 0.5° toward the (110) surface. If *τ* is sufficiently long for Ga diffusion to reach the step edges, Ga would be incorporated into the crystal at the step edges or kink sites. We found that the Ga surface lifetime before desorption above ~860 K was shorter than that before incorporation. This result suggests that the Ga adatom would desorb from the terrace because it could not reach the step edges or kink sites due to the short *τ*. Thus, the Ga incorporation–desorption transition temperature is estimated to be ~860 K, and this result agrees well with experimental results [66]. Therefore, the decrease of the GaAs growth rate becomes significant above ~920 K and suggests that our computational method is feasible for predicting the Ga surface lifetime, *τ*. As presented in Figure 7b, the calculated Ga diffusion coefficients are represented by brown solid lines with open squares for the $\left[1\bar{1}0\right]$ direction, $D_{\left[1\bar{1}0\right]}$, and with filled squares for the [110] direction, *D*_[110]_. The $D_{\left[1\bar{1}0\right]}$ is approximately five times larger than *D*_[110]_ because the Ga adatom easily migrates along the missing As-dimer rows along the $\left[1\bar{1}0\right]$ direction [67]. This result agrees with experimental results [68], and the Ga diffusion coefficient along the $\left[1\bar{1}0\right]$ direction is approximately four times larger than that along the [110] direction. In addition, the diffusion coefficient lines calculated as a function of reciprocal temperature all lie between the lines obtained by the experiments [65,69]. These results confirm the validity of our computational method for predicting *τ* and *D*.

**Table 3:** The migration barriers from *x* to *x*′ site, Δ*E*(*x*→*x*′), and desorption energies, *E*_de_(*x*), from the *x* site [67].

	*x*′	1	2	3	4	5	6	*E*_de_
*x*								
1			1.5	1.5	1.2	1.7	1.7	3.2
2		0.5		0.4	0.5	0.75	–	2.2
3		0.5	0.4		0.5	–	0.75	2.2
4		0.5	0.5	0.8		1.0	1.0	2.5
5		1.1	1.15	–	1.1		1.0	2.6
6		1.1	–	1.15	1.1	1.0		2.6

**Figure 8:**
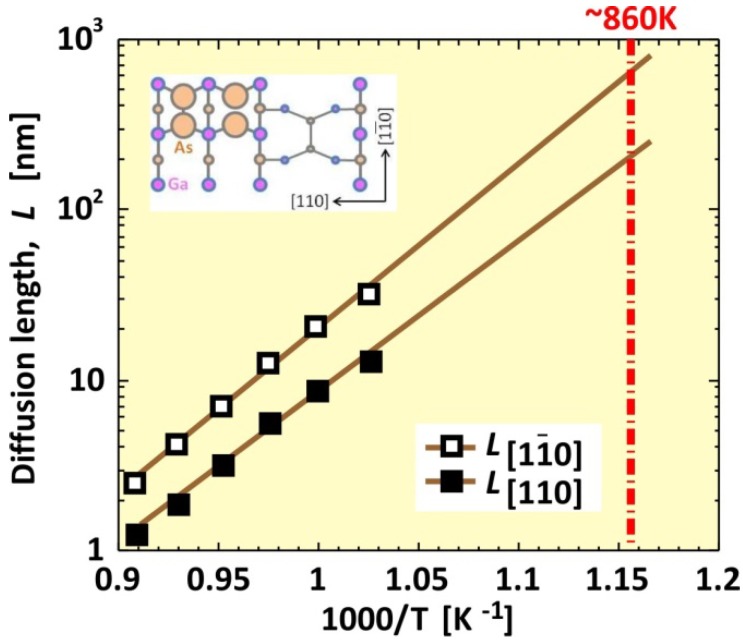
Ga diffusion length, *L*, as a function of reciprocal temperature at *p*_Ga_ = 1.4 × 10^−6^ Torr.

Next, we calculated the Ga diffusion length, *L*, on the -(2 × 4)β2 surface. Figure 8 shows *L* as a function of reciprocal temperature under the condition of *p*_Ga_ = 1.4 × 10^−6^ Torr. In Figure 8, solid lines with open and filled squares show the calculated Ga diffusion length along the $\left[1\bar{1}0\right]$ and [110] directions, respectively. The diffusion length decreases exponentially with temperature, even though the diffusion coefficient increases with temperature, as shown in Figure 7b. This behavior is because the Ga surface lifetime decrease influences the diffusion length more effectively than the influence of the diffusion coefficient increase. As presented in Figure 8, the extrapolated diffusion length value, $L_{\left[1\bar{1}0\right]}$, along the $\left[1\bar{1}0\right]$ direction is approximately 700 nm at the incorporation-desorption transition temperature (*T* = ~860 K). Figure 8 presents experiments from [70], where $L_{\left[1\bar{1}0\right]}$ = 250~1200 nm at 873 K. The results suggest that our computational method is appropriate for predicting the diffusion length on the surface.

## 4. Applications to III-Nitride Surfaces

The surface energy calculations based on the *ab initio* calculations for various surface structures have revealed that the stable surface reconstructions on III-nitride semiconductor surfaces are dependent on the chemical potential of constituent atomic species [7,23,51,52,71,72,73,74,75]. Although these *ab initio* studies successfully elucidated some aspects of the surface-related issues, their results do not include growth parameters, such as BEP and temperature. Thus, we applied an *ab initio*-based approach to the surface reconstructions and elemental growth processes on nitride semiconductors, which takes temperature and BEP into account. In this section, we present recent achievements that clarify the reconstruction, adsorption and incorporation on nitride semiconductor surfaces, including polar, non-polar and semipolar orientations, using this approach [34,35,36,37,38,39,40,41,42,43,44,45,46,47,48,49,50]. Surface phase diagram calculations as a function of temperature and BEP were performed for AlN, GaN, and InN surfaces with various orientations. The role of H adsorption was also investigated in conjunction with metal organic vapor phase epitaxy (MOVPE) growth in the surface phase diagram calculations. Additionally, Mg and C atom incorporation on the polar (0001) and semipolar $\left(1\bar{1}01\right)$ surfaces is systematically discussed using surface phase diagrams in terms of the contribution of hydrogen.

### 4.1. Surface Phase Diagram

#### 4.1.1. GaN Polar Surfaces

The reconstructed atomic structure during and after MBE growth on the GaN(0001) surface under Ga-rich conditions has been the subject of many experimental and theoretical investigations. The (2 × 2) and pseudo-(1 × 1) surfaces have been observed on GaN(0001) under Ga-rich conditions by STM [76,77]. Furthermore, the coexistence of a “ghost” island with the (2 × 2)-like structure and a normal island with the pseudo-(1 × 1) structure has been found under excess Ga fluxes [78]. There have been several *ab initio* theoretical studies for surface structures and adsorption behavior on these surfaces. Northrup *et al.* have proposed that among various surface structures, the pseudo-(1 × 1) structure is the most stable state under the Ga-rich limit [74]. Ishii investigated the stable adsorption behavior on the (2 × 2) structure under N- and Ga-rich conditions [75]. Although these *ab initio* studies have elucidated some aspects of the GaN surface, their results are limited to 0 K and did not incorporate growth parameters such as temperature and BEP.

**Figure 9:**
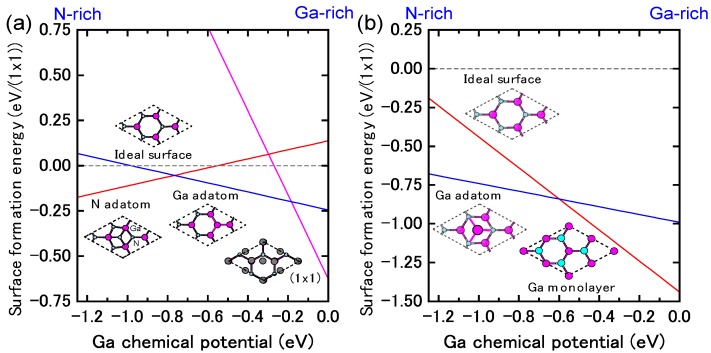
Calculated surface formation energies of polar GaN surfaces with (**a**) (0001) and (**b**) $\left(000\bar{1}\right)$ orientations as a function of Ga chemical potential. Schematics of the surface structures under consideration are also presented.

**Figure 10:**
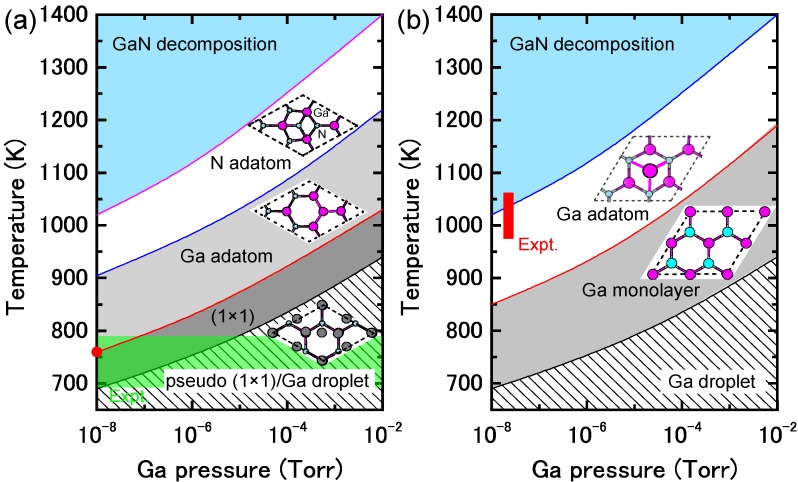
Calculated phase diagrams for polar GaN surfaces with (**a**) (0001) and (**b**) $\left(000\bar{1}\right)$ orientations as a function of temperature and Ga beam equivalent pressure (BEP). The stable reconstructions on these surfaces are also schematically presented. The shaded area denotes the molecular beam epitaxy (MBE) growth temperature range from Reference [78].

Figure 9a presents the calculated surface formation energy of GaN(0001) surfaces as a function of the Ga chemical potential using Equation (6). Here, the reconstructions considered are constructed on the basis of the electron counting (EC) rule [79], in which dangling bonds of the topmost Ga and N atoms are empty and filled by electrons, respectively. To satisfy the EC rule, the surface must be stabilized due to its semiconducting nature. In addition, the surfaces covered by Ga atoms are also considered to determine the stability under Ga-rich (high *μ*_Ga_) conditions. This energy diagram allows us to determine which reconstruction is the most stable. However, the reconstruction under growth conditions cannot be directly determined by this energy diagram. On the contrary, the surface diagram can be directly compared with the experiments because it is described as a function of the experimental parameters, such as temperature and BEP. Figure 10a presents the calculated phase diagram of the GaN(0001) surfaces as a function of temperature and Ga BEP [35,36,47]. The boundary lines separating different regions correspond to temperature and BEP in which two structures have the same formation energy. The stable reconstructions on these surfaces are also schematically presented in Figure 10. The pseudo-(1 × 1) surface is stable in the temperature range below 684 K at 10^−8^ Torr and below 973 K at 10^−2^ Torr. This stability is qualitatively consistent with the experimental stable temperature range for the pseudo-(1 × 1) surface [80]. The structure with additional Ga adatoms between the (1 × 1) and (2 × 2)-Ga structures does not appear to be a stable GaN(0001) structure because the Ga adsorption energy remains almost constant (2.6–2.8 eV) regardless of Ga coverage. Figure 10 also reveals that the (2 × 2) with the Ga adatom is stable in the temperature range of 767–1017 K at 10^−8^ Torr and 1078–1420 K at 10^−2^ Torr. These temperature ranges are consistent with the experimental stable temperature range for the (2 × 2) surface with Ga adatoms [81]. The Ga-vacant (2 × 2) structure is favorable for lower Ga BEP and higher temperatures because Ga desorption is enhanced at lower Ga BEP and higher temperatures. In addition, the ideal (cleaved and unrelaxed) surface does not appear in the phase diagram because the ideal surface does not satisfy the EC rule [79]. The (2 × 2) surface directly changes its structure from the (2 × 2) with Ga adatom to the (2 × 2) with Ga vacancy at lower Ga BEP and at higher temperatures.

From an experimental perspective, the (2 × 2) surface is often observed following an interruption in the Ga flux [82]. The phase diagram in Figure 10a qualitatively agrees with this experimental finding because a decrease in Ga BEP prefers the (2 × 2) surface with the Ga adatom to the pseudo-(1 × 1) and (1 × 1) surfaces at a certain temperature (e.g., ~800 K). The shaded area in Figure 10a denotes the temperature range for submonolayer GaN deposition. This temperature range includes the stable regions of the pseudo-(1 × 1), (1 × 1), and (2 × 2)-Ga surfaces. Thus, these results suggest that Ga adsorption or desorption can easily change the pseudo-(1 × 1) or (1 × 1) to the (2 × 2)-Ga surface and vice versa, depending on Ga BEP. This is also consistent with the STM observations [78].

The atomic structure of the reconstructions during and after MBE growth on the GaN$\left(000\bar{1}\right)$ surface under Ga-rich conditions has been studied by experimental and theoretical investigations [83]. The STM observations have clarified that the surface exhibits a (1 × 1) structure, and depositing additional Ga atoms onto this surface results in the (3 × 3), (6 × 6) and c(6 × 12) reconstructions. Based on *ab initio* calculations, it was determined that the (1 × 1) structure consists of a monolayer of Ga atoms bonded at the upper most sites above the topmost N atoms of an N-terminated bilayer. The (3 × 3) reconstruction consists of Ga adatoms bonded on top of this adlayer.

The calculated surface formation energy as a function of Ga chemical potential for a GaN$\left(000\bar{1}\right)$ surface is presented in Figure 9b. The results suggest that the surfaces with Ga adatoms and a Ga monolayer can be stabilized. However, as mentioned previously, the reconstruction under growth conditions cannot be directly determined. Figure 10b presents the calculated phase diagram of the GaN$\left(000\bar{1}\right)$ surfaces as a function of temperature and Ga BEP. The (2 × 2) surface with Ga adatoms is stabilized below 850 K at 10^−8^ Torr and below 1190 K at 10^−2^ Torr. On the contrary, the (1 × 1) surface that has a monolayer of Ga atoms is stable beyond 850 K at 10^−8^ Torr and 1190 K at 10^−2^ Torr. The surface phase diagram suggests that both surfaces can form at experimental temperatures (~1070 K), and the (1 × 1) surface with a monolayer of Ga atoms is favorable under Ga-rich conditions. Because the MBE on the GaN$\left(000\bar{1}\right)$ surface has been performed under Ga-rich conditions, the calculated result is qualitatively consistent with the experimental stable temperature range for the (1 × 1) surface [82]. In addition, the ideal surface does not appear in the phase diagram because the ideal surface does not satisfy the EC rule [79].

#### 4.1.2. GaN Nonpolar Surfaces

**Figure 11:**
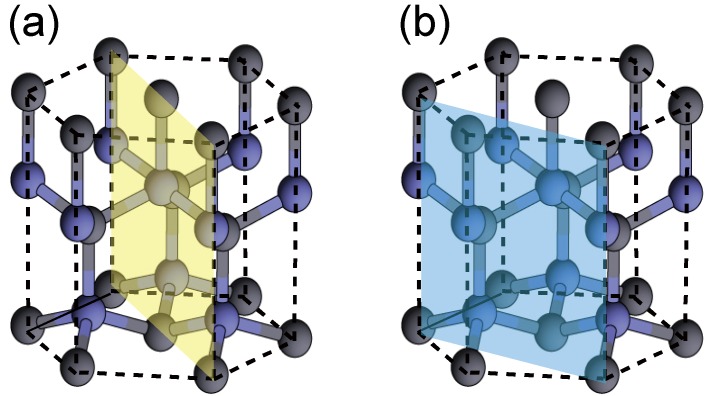
Schematics of crystal planes with nonpolar (**a**) $\left(1\bar{1}00\right)$ and (**b**) $\left(11\bar{2}0\right)$ orientations.

Epitaxial film growth has traditionally been performed along the polar [0001] direction, resulting in large polarization fields [84] along the growth direction. These fields reduce the radiative efficiency of quantum-well light emitters because they cause electron and hole separation. For optoelectronic device fabrication, there has been an increase in interest in the growth along nonpolar orientations, such as $\left(1\bar{1}00\right)$ and $\left(11\bar{2}0\right)$ planes, as presented in Figure 11a,b, respectively [85,86]. Previous *ab initio* calculations have determined that the ideal surface is most stable over a large range of chemical potentials and that surfaces with Ga adlayers are stabilized for Ga-rich conditions [87,88].

**Figure 12:**
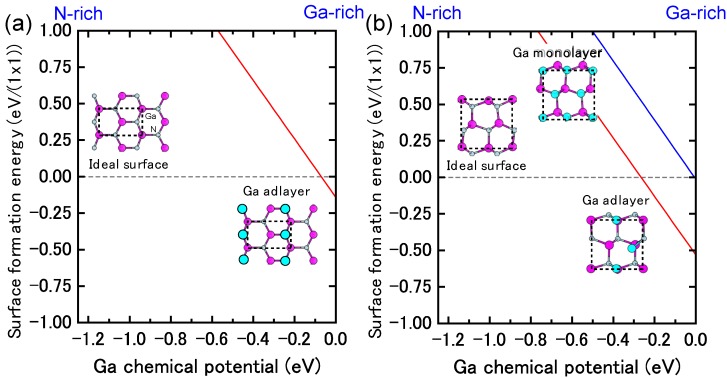
Calculated surface formation energies of nonpolar GaN surfaces with (**a**) $\left(1\bar{1}00\right)$ and (**b**) $\left(11\bar{2}0\right)$ orientations as a function of Ga chemical potential. Schematics of the surface structures under consideration are also presented.

**Figure 13:**
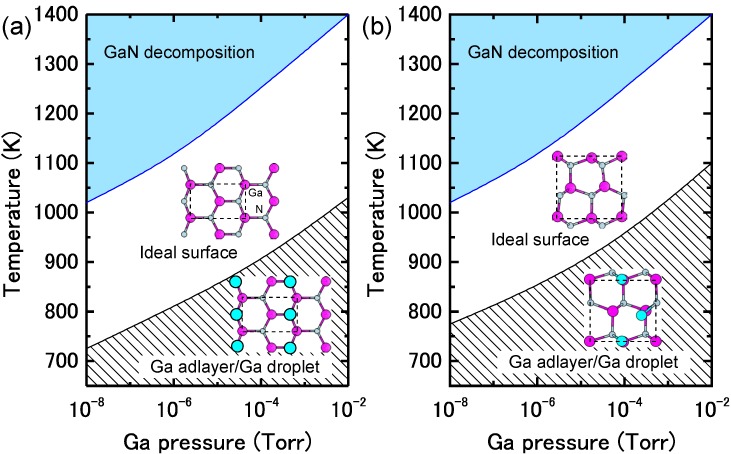
Calculated phase diagrams for nonpolar GaN surfaces with (**a**) $\left(1\bar{1}00\right)$ and (**b**) $\left(11\bar{2}0\right)$ orientations as a function of temperature and Ga BEP. The stable reconstructions on these surfaces are also schematically presented.

The reconstructions on nonpolar $\left(1\bar{1}00\right)$ and $\left(11\bar{2}0\right)$ surfaces are very simple, as presented in Figure 12 and Figure 13 [45,47]. The calculated surface formation energies presented in Figure 12 demonstrate that the ideal surface is stabilized over a wide Ga chemical potential range. In contrast, the calculated surface phase diagrams presented in Figure 13 suggest that the ideal surface appears beyond the temperature range of 725–1030 K and 770–1080 K on $\left(1\bar{1}00\right)$ and $\left(11\bar{2}0\right)$ surfaces, respectively. However, the Ga adlayer surfaces are stable only at lower temperatures. For the ideal surfaces, the N atom relaxes outward whereas the Ga atom relaxes inward, which is accompanied by a charge transfer from the Ga dangling bond to the N dangling bond. As a result of this charge transfer, the ideal surface satisfies the EC rule [79] and is stabilized without any adsorption or desorption to the surface. Therefore, the MBE growth proceeds on the ideal GaN$\left(11\bar{2}0\right)$ surface regardless of Ga BEP at the conventional growth temperatures.

#### 4.1.3. GaN Semipolar Surfaces

**Figure 14:**
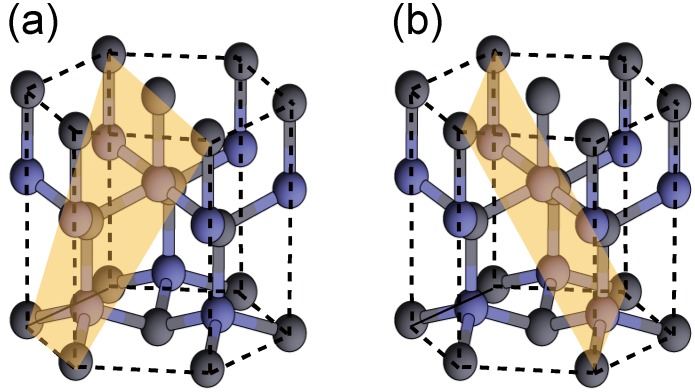
Crystal plane schematics for semipolar (**a**) $\left(1\bar{1}01\right)$ and (**b**) $\left(11\bar{2}2\right)$ orientations.

In addition to nonpolar orientations, there is an increasing interest in crystal growth and device fabrication on semipolar orientations, such as $\left(1\bar{1}01\right)$ and $\left(11\bar{2}2\right)$, as presented in Figure 14a,b, respectively, due to their reduced or negligible electric field [89,90,91,92,93,94,95]. A recent report concluded that one Ga monolayer can be stabilized on a GaN$\left(11\bar{2}2\right)$ surface when deposited under Ga-rich conditions using plasma-assisted molecular beam epitaxy (MBE). The GaN$\left(11\bar{2}2\right)$surface was necessary to optimize the surface morphology [96].

**Figure 15:**
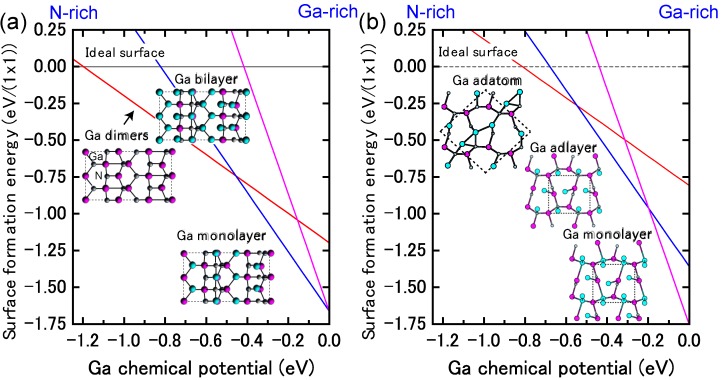
Calculated surface formation energies for semipolar GaN surfaces with (**a**) $\left(1\bar{1}01\right)$ and (**b**) $\left(11\bar{2}2\right)$ orientations as a function of the Ga chemical potential. Schematics of the surface structures under consideration are also presented.

**Figure 16:**
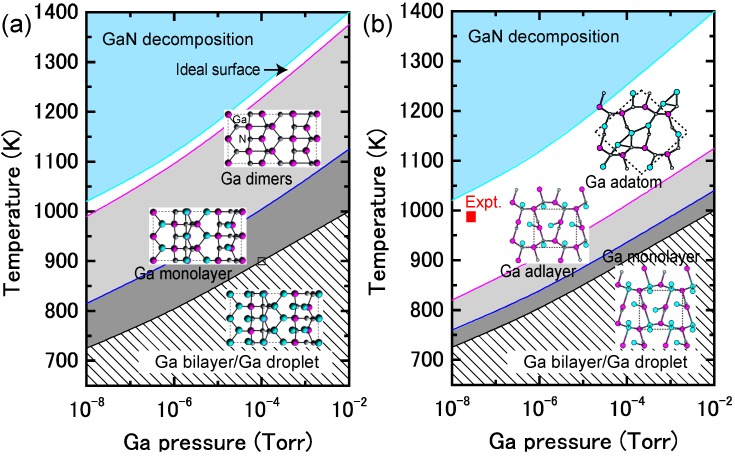
Calculated phase diagrams for nonpolar GaN surfaces with (**a**) $\left(1\bar{1}01\right)$ and (**b**) $\left(11\bar{2}2\right)$ orientations as a function of temperature and Ga BEP. The surface stable reconstructions are also schematically presented.

Figure 15a displays the calculated surface formation energies of a semipolar GaN$\left(1\bar{1}01\right)$ surface, demonstrating that many reconstruction types can be used depending on the Ga chemical potential. The surfaces that have Ga atoms at the topmost layer are stabilized over a wide range of Ga chemical potentials. The semipolar GaN$\left(1\bar{1}01\right)$ surface phase diagram is presented in Figure 16a [44]. With increasing temperature, the Ga bilayer metallic reconstruction that is stabilized at low temperatures changes its structure from a Ga monolayer to Ga dimers. The metallic reconstruction was stabilized under Ga-rich conditions similarly to the GaN(0001) surface. Therefore, many types of reconstructions could appear at approximately 1100 K (a typical MBE growth temperature) depending on the Ga BEP, even though the stabilization temperature range for the ideal surface is very narrow. This conclusion suggests that the GaN$\left(1\bar{1}01\right)$ surface growth kinetics depend on the growth temperatures.

The calculated surface formation energy for GaN$\left(11\bar{2}2\right)$ is displayed in Figure 15b and suggests that several reconstructions can occur depending on the Ga chemical potential. The metallic reconstructions that have a Ga adlayer or monolayer are stabilized under Ga-rich conditions; however, the Ga adatom surface is favored under N-rich conditions. Figure 16b presents the semipolar GaN$\left(11\bar{2}2\right)$surface phase diagram [41]. The diagram suggests that the metallic reconstructions with a Ga adlayer or a monolayer emerge only at low temperatures and high Ga-rich conditions. In contrast, the Ga adatom surface is favored over a wide temperature range. The calculated surface phase diagram agrees well with the experimental results in which the Ga monolayer surface was formed under high Ga fluxes near the Ga accumulation (droplet) onset in the plasma-assisted MBE (*T* ~ 1000 K) [96]. The calculated phase diagram thus suggests that the GaN$\left(11\bar{2}2\right)$ surface growth kinetics are similar to the GaN$\left(1\bar{1}01\right)$ surface and depend on the growth temperatures.

#### 4.1.4. InN Polar Surfaces

High quality InN is known to be difficult to grow compared with other III-nitrides, such as AlN and GaN, because of a relatively low dissociation temperature and a high equilibrium N_2_ vapor pressure [97,98]. Nevertheless, several researchers [99,100,101] have successfully grown high-quality InN crystals. These reports suggest that polarity termination is an important consideration when growing high-quality group-III nitride semiconductors [102]. The InN radio frequency MBE growth on a sapphire substrate [102] produced surfaces with varying polarities depending on the growth temperature. Thus, the atomic structures of the In surface reconstructions have been the subject of many experimental and theoretical investigations. Gan and Srolovitz have proposed several stable structures on InN(0001) depending on the growth conditions, whereas an In monolayer directly above the surface N atoms is stabilized on a InN$\left(000\bar{1}\right)$ surface [87,103]. However, few comparisons exist between experiments and theoretical calculations.

**Figure 17:**
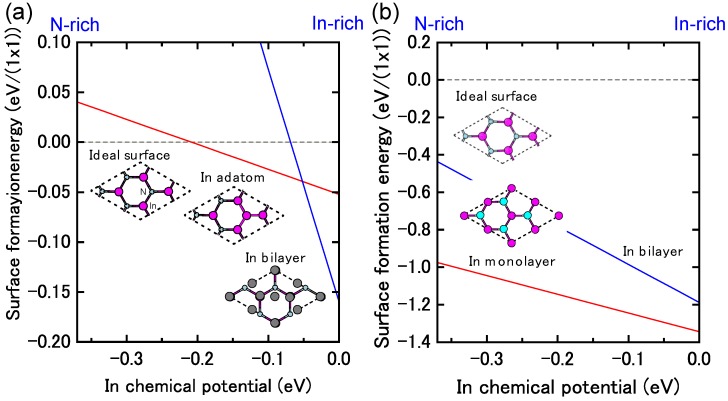
Calculated surface formation energies of polar InN surfaces with (**a**) (0001) and (**b**) $\left(000\bar{1}\right)$ orientations as a function of In chemical potential. Schematics of the surface structures under consideration are also presented.

**Figure 18:**
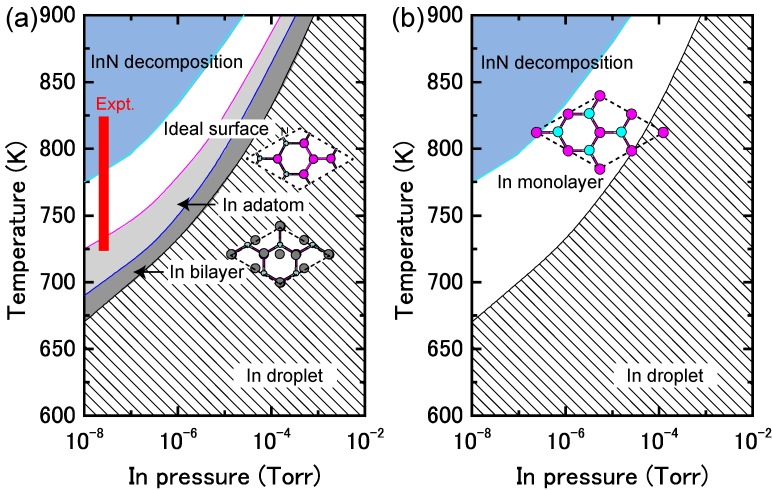
Calculated phase diagrams for polar InN surfaces with (**a**) (0001) and (**b**) $\left(000\bar{1}\right)$ orientations as a function of temperature and In BEP. The surface stable reconstructions are also schematically presented.

Figure 17a displays the calculated surface formation energy for InN(0001) surfaces as a function of the In chemical potential. There are three types of reconstructions including the ideal surface and depending on the In chemical potential. The metallic surface with an In bilayer is stabilized under extremely In-rich conditions. As shown in Figure 18a, the calculated surface phase diagram of InN(0001) indicates that this metallic surface is stable in the temperature range below 695 K at 10^−8^ Torr and below 850 K at 10^−4^ Torr. The (2 × 2) surface with an In adatom is stabilized under the moderate conditions of 695–720 K at 10^−8^ Torr and 860–900 K at 10^−4^ Torr. The ideal surface is favored for lower In BEP and higher temperatures because In desorption is enhanced at lower In BEP and higher temperatures. Therefore, during the MBE growth of InN (725–825 K), the surface changes from the In bilayer metallic surface to an ideal In adatom surface via the (2 × 2) structure at a lower In BEP and higher temperatures. Although experimental data are not available for comparison to the calculated results, the theoretical trend reasonably agrees with previous *ab initio* calculations. The In bilayer and the ideal surface are both stabilized, even though they do not satisfy the electron EC rule [79]. These stable (0001) surface structures differ from those found for other closely related group-III nitrides, such as AlN and GaN, over the entire temperature and pressure range.

On the contrary, the calculated surface formation energy of an InN$\left(000\bar{1}\right)$ surface suggests that there is only one reconstruction over the entire range of In chemical potentials. The In monolayer surface is always stable, which is shown by the surface phase diagram of the InN$\left(000\bar{1}\right)$ surface that is presented in Figure 18b. The phase diagram suggests that the In monolayer surface is stabilized regardless of the growth conditions, which is different from the GaN$\left(000\bar{1}\right)$ surfaces. In GaN surfaces, the lowest energy surface structure is a Ga monolayer and occurs only at low temperatures. However, under N-rich conditions, the GaN$\left(000\bar{1}\right)$ surface with a Ga adatom on the H3 site in the (2 × 2) unit cell has the lowest energy, as shown in Figure 18b. The In monolayer stabilization on an InN$\left(000\bar{1}\right)$ surface could be related to the interatomic distances between the In monolayer (~3.16 Å) atoms, which are similar to the tetragonal symmetrical bulk In (3.28 Å) distances.

#### 4.1.5. InN Nonpolar Surfaces

**Figure 19:**
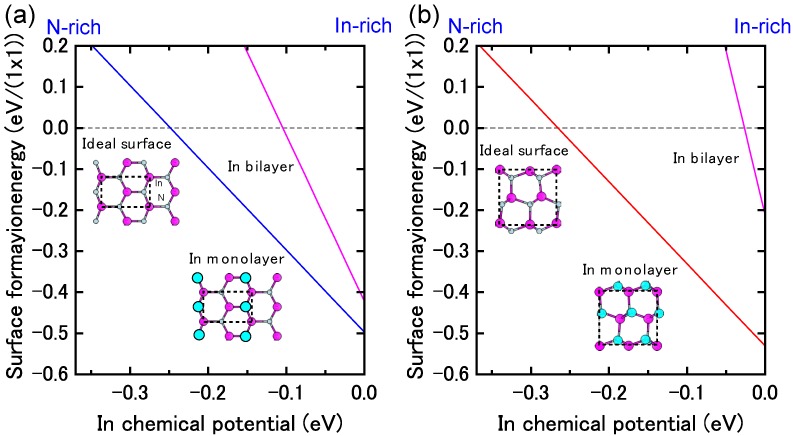
Calculated surface formation energies for nonpolar InN surfaces with (**a**) $\left(1\bar{1}00\right)$ and (**b**) $\left(11\bar{2}0\right)$ orientations as a function of the In chemical potential. Schematics of the surface structures under consideration are also presented.

**Figure 20:**
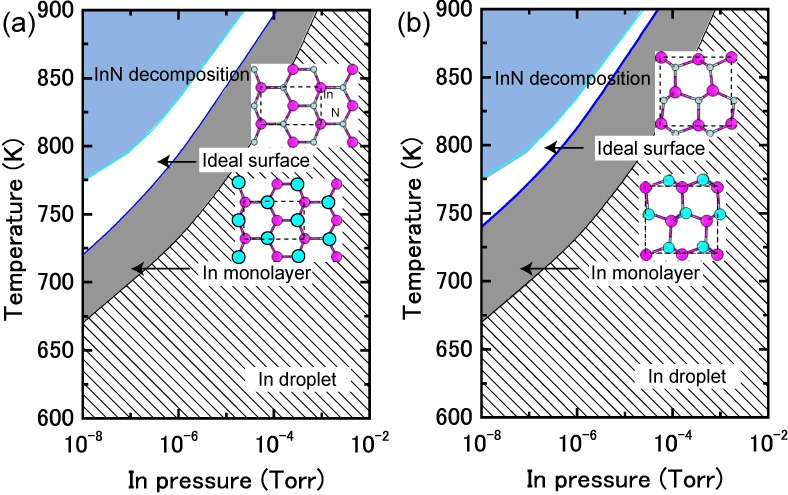
Calculated phase diagrams for nonpolar InN surfaces with (**a**) $\left(1\bar{1}00\right)$ and (**b**) $\left(11\bar{2}0\right)$ orientations as a function of temperature and In BEP. The stable reconstructions on these surfaces are also schematically presented.

Figure 19 presents the calculated surface formation energies for InN nonpolar surfaces as a function of the In chemical potential. The results are consistent with previous *ab initio* calculations, which have suggested that the InN *nonpolar* planes are similar to GaN. In-N dimers are stable at moderate and high In/N ratios, and the metallic reconstructions are similar to those in GaN surfaces [38,87]. The calculated surface phase diagrams for nonpolar InN surfaces, presented in Figure 20, successfully reproduce these structural characteristics depending on the growth conditions [45]. The ideal surface appears beyond the temperature range of 720–1010 K and 740–1035 K on $\left(1\bar{1}00\right)$ and $\left(11\bar{2}0\right)$ surfaces, respectively, whereas the surfaces with an In monolayer are stable at lower temperatures. For the ideal surfaces, the N atom relaxes outward similar to the GaN nonpolar surfaces, whereas the In atom relaxes inward and is accompanied by a charge transfer from the In dangling bond to the N dangling bond. The ideal surface thus satisfies the EC rule [79] and is stabilized without any adsorption or desorption to the surface. As expected for nonpolar orientations, the InN MBE growth of an In monolayer proceeds over a wide range of In BEP and at the conventional growth temperatures (725–825 K).

#### 4.1.6. InN Semipolar Surfaces

**Figure 21:**
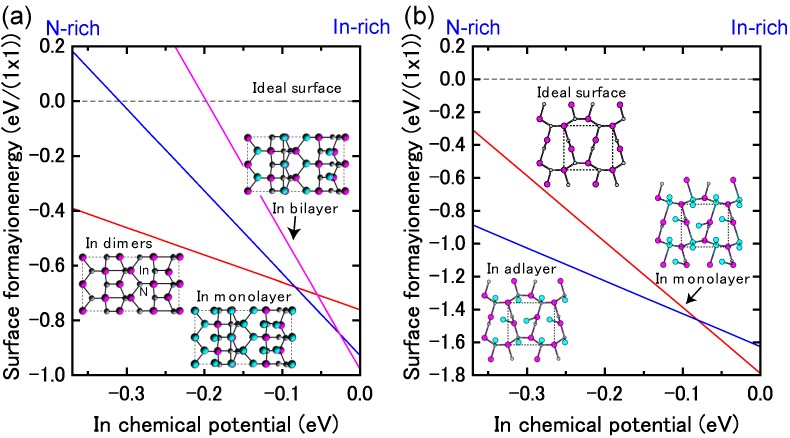
Calculated surface formation energies for semipolar InN surfaces with (**a**) $\left(1\bar{1}01\right)$ and (**b**) $\left(11\bar{2}2\right)$ orientations as a function of In chemical potential. Schematics of the surface structures under consideration are also presented.

**Figure 22:**
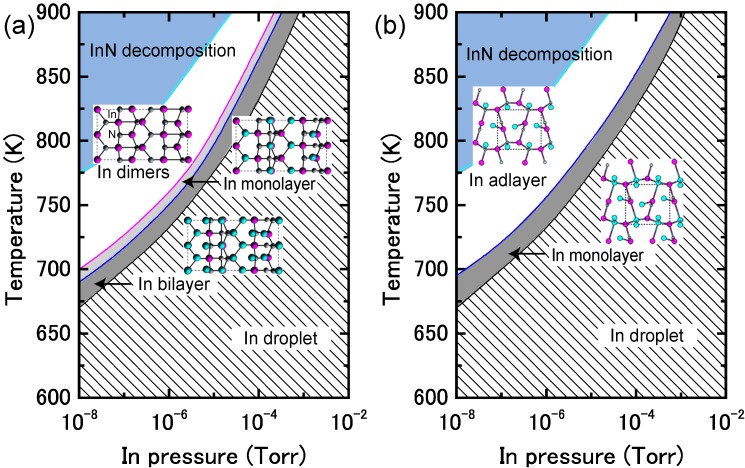
Calculated phase diagrams for nonpolar InN surfaces with (**a**) $\left(1\bar{1}01\right)$ and (**b**) $\left(11\bar{2}2\right)$ orientations as a function of temperature and In BEP. The stable reconstructions on these surfaces are also schematically presented.

Figure 21 presents the calculated surface formation energies for InN semipolar surfaces as a function of the In chemical potential. The InN stable surfaces differ from the semipolar GaN surfaces. Indeed, the calculated InN semipolar surface phase diagrams, presented in Figure 22, are slightly different from the semipolar GaN surfaces because of the narrow growth chemical potential range of InN [45]. For the InN$\left(1\bar{1}01\right)$ surface presented in Figure 22a, there are several possible reconstructions depending on temperature and In BEP. The metallic reconstruction with the In bilayer that is stabilized at low temperatures changes its In monolayer structure to contain In dimers upon higher temperatures. The metallic reconstruction stabilization under an In-rich condition is similar to what occurs for the GaN$\left(1\bar{1}01\right)$ surface. Therefore, many reconstruction types can appear during the MBE growth depending on the In BEP, which also suggests that the InN$\left(1\bar{1}01\right)$ surface growth kinetics depend on the growth temperatures.

The semipolar InN$\left(11\bar{2}2\right)$ surface phase diagram presented in Figure 22b suggests that the metallic reconstruction with an In monolayer emerges only at low temperatures and in In-rich conditions [45]. In contrast, the In adlayer surface is favored over the wide temperature range. The calculated phase diagram thus suggests that the MBE growth proceeds on the In adlayer surface over a wide range of In BEP. Although few experimental reports have been published for the structure of InN semipolar surfaces, our results provide a firm theoretical framework for predicting the InN$\left(11\bar{2}2\right)$ surface reconstructions.

#### 4.1.7. AlN Polar Surfaces

Because AlN has the largest band gap among the group-III nitride semiconductors, AlN materials have attracted attention for high-power and high-temperature ultraviolet photoelectronic devices. Thus far, the growth of high-quality AlN layers has been accomplished using epitaxial growth techniques, such as MBE [104,105,106], MOVPE [107,108,109] and hydride vapor-phase epitaxy [110,111]. Control over the growth conditions is one important factor for fabricating high-quality crystals and could be achieved through an understanding of surface reconstructions. Therefore, many theoretical studies on AlN surface reconstructions have been reported, and different reconstructions have been found depending on the growth conditions [105,112,113,114,115,116].

**Figure 23:**
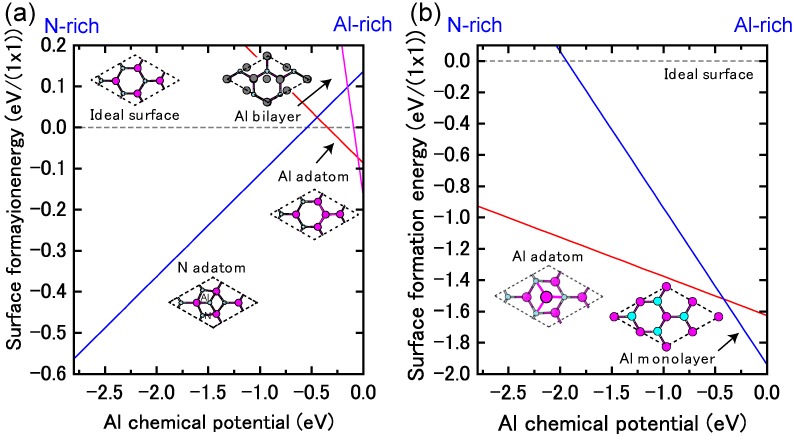
Calculated surface formation energies for polar AlN surfaces with (**a**) (0001) and (**b**) $\left(000\bar{1}\right)$ orientations as a function of Al chemical potential. Schematics of the surface structures under consideration are also presented.

**Figure 24:**
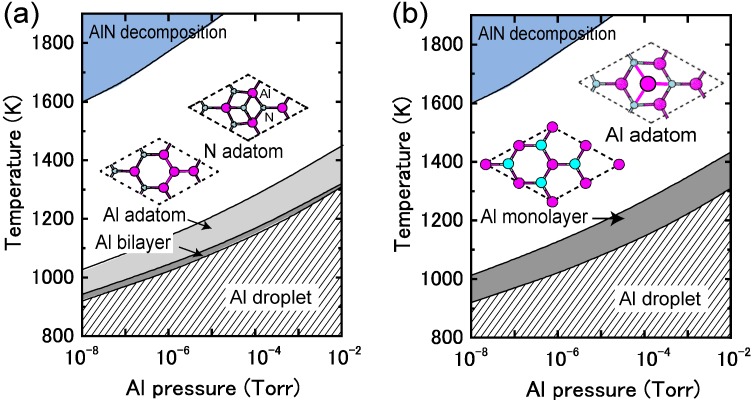
Calculated phase diagrams for polar AlN surfaces with (**a**) (0001) and (**b**) $\left(000\bar{1}\right)$ orientations as a function of temperature and Al BEP. The stable reconstructions on these surfaces are also schematically presented.

Figure 23 displays the calculated surface formation energies for AlN polar surfaces as a function of the Al chemical potential. These formation energies indicate that the stable AlN polar surface structures are slightly different from those on GaN surfaces. Furthermore, the calculated AlN polar surface phase diagrams presented in Figure 24 are slightly different from the GaN surface because of the wide growth chemical potential range of GaN. The calculated AlN(0001) surface phase diagram presented in Figure 24a suggests that the Al metallic bilayer surface is stable in a narrow temperature range below 940 K at 10^−8^ Torr and below 1320 K at 10^−2^ Torr. This figure also reveals that the (2 × 2) Al adatom surface is stable at 940–1030 K at 10^−8^ Torr and at 1320–1455 K at 10^−2^ Torr. The N adatom surface is favorable at lower Al BEP and higher temperatures because Al desorption is enhanced at these conditions. The Al bilayer surface is stabilized even though it does not satisfy the EC rule. Due to a wide growth chemical potential range (2.78 eV), the stable region for the N adatom on the AlN surface is considerably larger than for the GaN surface.

Figure 24b displays the calculated AlN$\left(000\bar{1}\right)$ surface phase diagram as a function of temperature and Al BEP. The (2 × 2) Al adatom surface is stabilized below 1020 K at 10^−8^ Torr and below 1440 K at 10^−2^ Torr. However, the (1 × 1) surface with a monolayer of Al atoms is stable above 1020 K at 10^−8^ Torr and above 1440 K at 10^−2^ Torr. The surface phase diagram suggests that both surfaces can be obtained at an AlN MBE growth experimental temperature of ~1200 K, and the Al monolayer (1 × 1) surface is favorable under Al-rich conditions.

**Figure 25:**
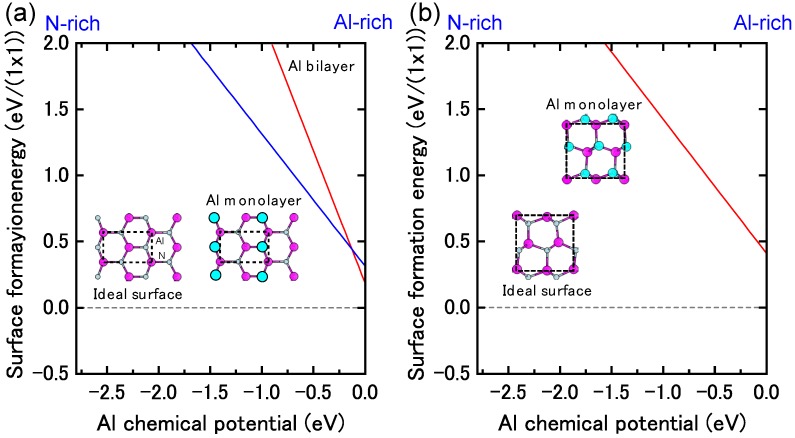
Calculated surface formation energies for polar AlN surfaces with (**a**) $\left(1\bar{1}00\right)$ and (**b**) $\left(11\bar{2}0\right)$ orientations as a function of Al chemical potential. Schematics of the surface structures under consideration are also presented.

#### 4.1.8. AlN Non-polar Surfaces

**Figure 26:**
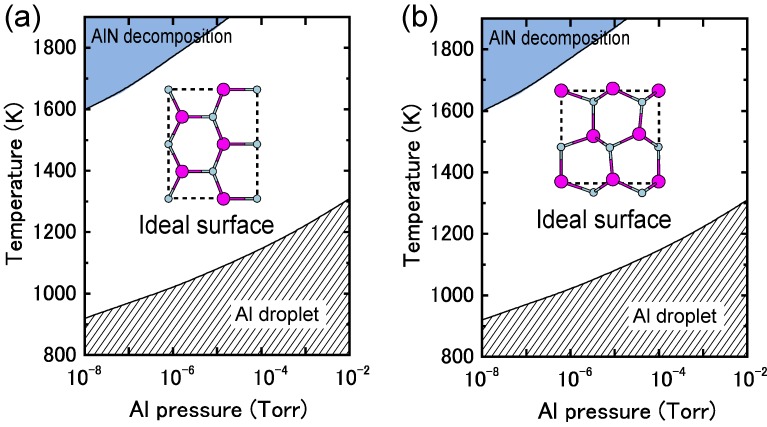
Calculated phase diagrams for nonpolar AlN surfaces with (**a**) $\left(1\bar{1}00\right)$ and (**b**) $\left(11\bar{2}0\right)$ orientations as a function of temperature and Al BEP. The stable reconstructions on these surfaces are also schematically presented.

Figure 25 presents the calculated AlN nonpolar surface formation energies as a function of the Al chemical potential. These results are consistent with previous *ab initio* calculations that have suggested that AlN *nonpolar* planes are slightly different from GaN surfaces. Al-N dimers are stable over a wide range of growth conditions [116]. The calculated nonpolar AlN surface phase diagrams presented in Figure 26 successfully reproduce the stability of the AlN nonpolar surface regardless of the growth conditions. The ideal surface appears over the entire temperature range. The Al bilayer and monolayer surfaces are always metastable. For the ideal surfaces, the N atom relaxes outward similar to the GaN nonpolar surfaces, whereas the In atom relaxes inward and is accompanied by a charge transfer from the Al dangling bond to the N dangling bond. The ideal surface thus satisfies the EC rule [79] and is stabilized without any adsorption or desorption in the surface. We thus concluded that the AlN MBE growth on nonpolar orientations proceeds on the ideal surface over the entire range of Al BEP.

### 4.2. Hydrogen Adsorption

#### 4.2.1. GaN Surfaces

**Figure 27:**
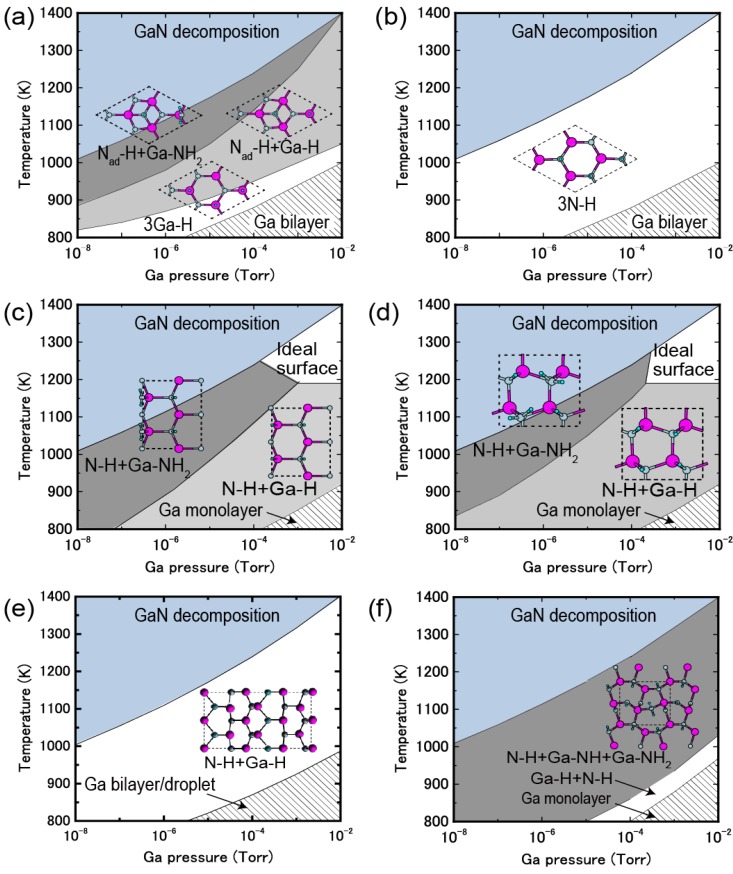
Calculated H-adsorbed GaN surface phase diagrams for polar (**a**) (0001) and (**b**) $\left(000\bar{1}\right)$; nonpolar (**c**) $\left(1\bar{1}00\right)$ and (**d**) $\left(11\bar{2}0\right)$; and semipolar (**e**) $\left(1\bar{1}01\right)$ and (**f**) $\left(11\bar{2}2\right)$ orientations as a function of temperature and Ga BEP under high H_2_ pressure (*p*_H2_ = 76 Torr) conditions. Top surface structure views are also presented. Large, small, and tiny circles represent Ga, N, and H atoms, respectively.

During MOVPE growth, the surface is exposed to high H_2_ pressures and interacts with H-rich ambient conditions. Under high H_2_ pressures during MOVPE growth, the stable structures differ from those exposed to low H_2_ pressures. Thus, clarifying the reconstruction and taking the hydrogen adsorption into account is indispensable when investigating the nitride surface stability during the MOVPE growth. Therefore, hydrogen stability was systematically investigated on various GaN surfaces. Figure 27 displays the calculated H-adsorbed GaN surface phase diagrams for polar (0001), polar $\left(000\bar{1}\right)$, nonpolar $\left(1\bar{1}00\right)$, nonpolar $\left(11\bar{2}0\right)$, semipolar $\left(1\bar{1}01\right)$ and semipolar $\left(11\bar{2}2\right)$ orientations as a function of temperature and Ga BEP [44,45,47]. The surface phase diagrams are obtained assuming the H_2_ pressure, *p*_H2 _= 76 Torr (0.1 atm), corresponds to H-rich conditions. The H atom adsorption exhibits a different surface phase diagram trend compared with those without H atoms, as presented in Figure 10, Figure 13 and Figure 16. The NH and NH_2_ H-terminated surfaces are typically formed over a wide range of temperatures and Ga BEP.

The polar GaN(0001) surface phase diagram presented in Figure 27a demonstrates that N_ad_–H + Ga–H can be formed from 1270 to 1370 K at *p*_Ga_ ≥ 10^−3^ Torr [45]. The diagram also indicates that N_ad_–H + Ga–H and N_ad_–H + Ga–NH_2_ are stabilized at low temperatures and high temperatures, respectively. Because both N_ad_–H + Ga–H and N_ad_–H + Ga–NH_2_ satisfy the EC rule [79], the stabilization of N_ad_–H + Ga–NH_2_ under N-rich conditions can be interpreted in terms of the desorption of Ga atoms. In N_ad_–H + Ga–H, the topmost Ga atoms desorb and N atoms appear with decreasing μ_Ga_. Because the surface is exposed to H-rich conditions, H atoms terminate the remaining N atoms, which results in the formation of H-terminated N adatoms and Ga–NH_2_ bonds.

For the GaN$\left(000\bar{1}\right)$ surfaces presented in Figure 27b, the surfaces terminated by H atoms (3N–H) were stabilized over the entire temperature and Ga BEP range. This suggests that the GaN$\left(000\bar{1}\right)$ surface reconstruction is insensitive to the growth conditions. The Ga adatom reconstructions that grow without H atoms that are presented in Figure 10b are not favorable because the N–N bond formation leads to N_2_ molecular desorption. Because the N–H bond has a very stable configuration among bonds between Ga, N and H atoms, the H atoms easily terminate the topmost N atoms and a large energy gain (~4 eV) occurs. The formation of three N–H bonds leads to a charge transfer from the N–H bond to the remaining N dangling bond, which results in the formation of filled dangling bonds (lone pairs) to satisfy the EC rule [79]. This structure corresponds to the strong affinity of hydrogen.

The nonpolar GaN $\left(1\bar{1}00\right)$ and $\left(11\bar{2}0\right)$ surfaces presented in Figure 27c, d, respectively, form NH_2_-terminated surfaces similar to the GaN(0001) surface [45,47]. However, the stable structures are different from those found on the GaN(0001) surface, depending on the growth temperature. The surface consists of Ga–N dimers, and the ideal surface presented in Figure 13 is stable even under the H-rich conditions at 1200–1400 K at *p*_Ga_ ≥ 10^−4^ Torr. This stability is because dangling bonds of the topmost Ga are empty, and the topmost N atoms are filled by electrons, which both satisfy the EC rule [79]. At *p*_Ga_ ≤ 10^−4^ Torr, N–H + Ga–NH_2_ is favorable below 1300 K. Thus, two different types of reconstructions can occur with MOVPE growth on nonpolar orientations.

In contrast to polar and nonpolar surfaces, the semipolar GaN$\left(1\bar{1}01\right)$ surface phase diagram is simple, as shown in Figure 27e [44]. The surface with N atoms at the top layer and also Ga atoms at the top layer with Ga–Ga dimers that are terminated by H atoms is stable over a wide range of Ga BEPs and temperatures suggesting that N–H + Ga–H usually appears during the MOVPE growth. The bonding states of the Ga–Ga dimer and Ga–H bonds are completely occupied by the excess electrons due to the N–H bonds. Thus, the 4N–H + Ga–H stabilization can be interpreted in terms of the EC rule [79], as observed for the H-adsorbed polar and nonpolar GaN surfaces. The GaN$\left(11\bar{2}2\right)$ surface phase diagram presented in Figure 27f indicates that the stable region of the N–H + Ga–NH + Ga–NH_2_ expands over the wide temperature and Ga BEP range, suggesting that this structure always emerges at temperatures ranging from 1200 to 1400 K regardless of Ga pressure [45,47]. This suggests that N–H + Ga–NH + Ga–NH_2_ will emerge during MOVPE regardless of the growth conditions. The surface stabilization is related to the Ga$\left(11\bar{2}2\right)$ surface polarity. The N-terminated surface where the two- and three-coordinated topmost N atoms appear is the ideal cleavage surface. Because the N–H bond is a very stable configuration and can form bonds with Ga, N and H atoms, the H atoms easily terminate the topmost N atoms with a large increase in energy (~4 eV). To satisfy the EC rule [79], three of the eight top N atoms have lone pairs. This structure is similar to the stable H-terminated GaN$\left(000\bar{1}\right)$ surface, which has a strong hydrogen affinity.

#### 4.2.2. InN Surfaces

In contrast, it is known that growth on InN is prevented with increasing H_2_ pressure and when N_2_ is used as the carrier gas. Thermodynamic analysis has also shown that the InN deposition rate decreases with increasing hydrogen pressure [117], suggesting that surface reconstructions and growth kinetics on InN surfaces are different than on GaN surfaces. From a theoretical perspective, the reconstructions on nonpolar and semipolar InN surfaces under different MBE growth conditions have been investigated, and several stable structures have been found depending on the growth conditions [38,39,81,99]. However, hydrogen stability and temperature and pressure dependence on InN surfaces have been less reported than for clean InN surfaces.

**Figure 28:**
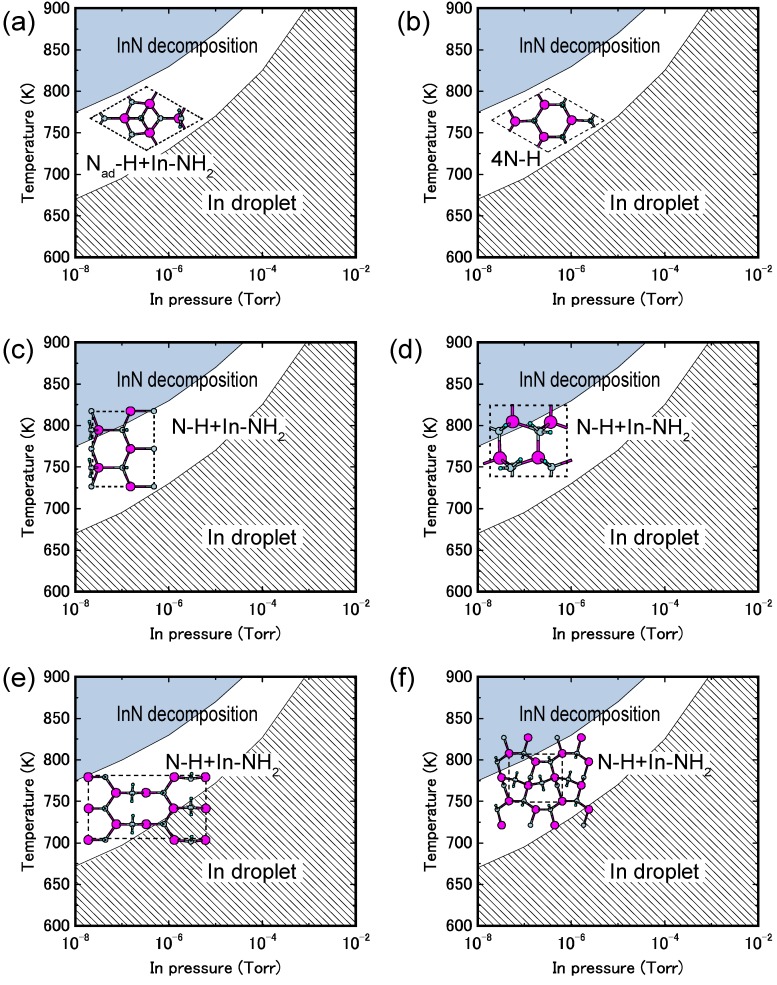
Calculated H-adsorbed InN surface phase diagrams for polar (**a**) (0001) and (**b**) $\left(000\bar{1}\right)$, nonpolar (**c**) $\left(1\bar{1}00\right)$ and (**d**) $\left(11\bar{2}0\right)$, and semipolar (**e**) $\left(1\bar{1}01\right)$ and (**f**) $\left(1\bar{1}22\right)$ orientations as a function of temperature and In BEP under high H_2_ pressure (*p*_H2_ = 76 Torr) conditions. Top surface structure views are also presented. Large, small, and tiny circles represent In, N, and H atoms, respectively.

Figure 28 displays the H-adsorbed InN surface phase diagrams for polar (0001), polar $\left(000\bar{1}\right)$, nonpolar $\left(1\bar{1}00\right)$, nonpolar $\left(11\bar{2}0\right)$, semipolar $\left(1\bar{1}01\right)$ and semipolar $\left(11\bar{2}2\right)$ orientations as a function of temperature and In BEP, assuming an H_2_ pressure (*p*_H2_ = 76 Torr) that corresponds to H-rich conditions [45]. Similar to GaN surfaces, H atom adsorption exhibits a different surface phase diagram compared with those without H atoms, as presented in Figure 18, Figure 20 and Figure 22. These surface phase diagrams demonstrate that the N–H and NH_2_ H-terminated surfaces are stabilized at temperatures above 675–900 K. Therefore, the H-terminated surfaces, such as the N_ad_–H + In–NH_2_ on the InN(0001) surface (Figure 28a), the 4N–H on the InN$\left(000\bar{1}\right)$ surface (Figure 28b), and the N–H + In–NH_2_ on the InN$\left(1\bar{1}00\right)$, $\left(11\bar{2}0\right)$, $\left(1\bar{1}01\right)$, and $\left(11\bar{2}2\right)$ surfaces (Figure 28c–f, respectively), always emerge regardless of the growth conditions. Because there are excess electrons on these surfaces, the InN surface stability of nonpolar and semipolar orientations is quite different from GaN surfaces. Because of low InN growth temperatures, the surfaces with a large number of N–H bonds become the most favorable configurations even though many excess electrons are generated by N–H bonds. The EC rule [79] is no longer satisfied on semipolar surfaces. The absence of orientation dependence suggests that the growth kinetics on nonpolar and semipolar surfaces are similar to polar surfaces. Because the growth of InN on a InN(0001) surface is known to be prevented at high H_2_ pressures, the growth on nonpolar and semipolar surfaces is also inhibited due to the presence of hydrogen. Although the adsorption and desorption behavior of In and N on the H-terminated surfaces with NH_2_ should be verified, the desorption of In and N atoms on these surfaces likely easily occurs.

#### 4.2.3. AlN Surfaces

**Figure 29:**
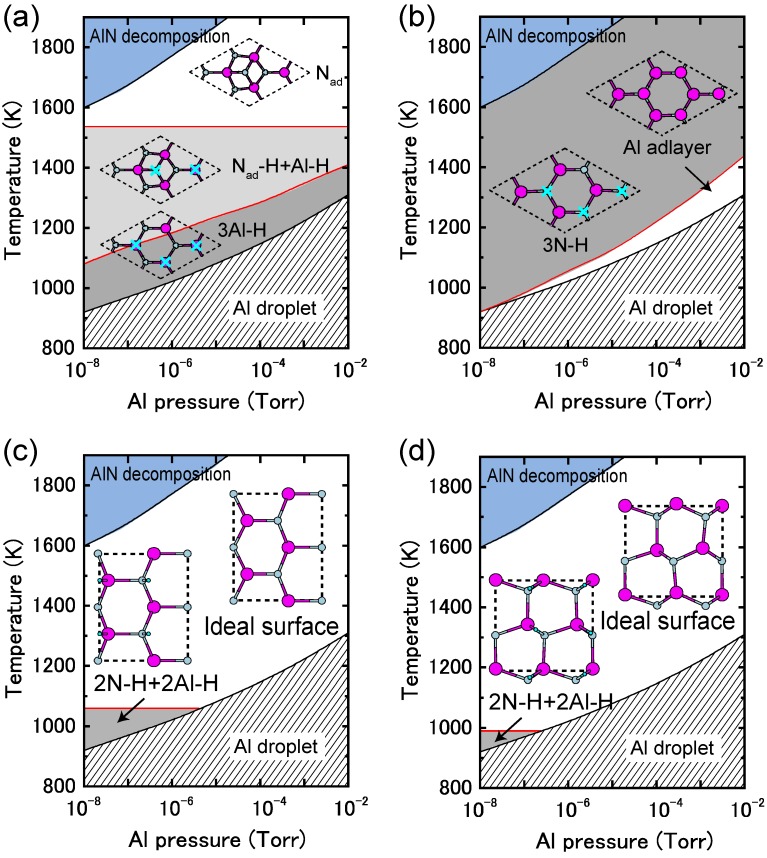
Calculated H-adsorbed AlN surface phase diagrams for polar (**a**) (0001) and (**b**) $\left(000\bar{1}\right)$ and nonpolar (**c**) $\left(1\bar{1}00\right)$ and (**d**) $\left(11\bar{2}0\right)$ as a function of temperature and In BEP under high H_2_ pressure (*p*_H2_ = 76 Torr) conditions. Top surface structure views are also presented. Large and small circles represent Al and N atoms, respectively. The positions of H atoms in the H-terminated surfaces are marked by crosses.

Although many theoretical studies on AlN surface reconstructions have been reported [79,112,113,114,115,116], most did not take into account the presence of H atoms Compared to the H adsorption on GaN surfaces, H atom adsorption on AlN polar and nonpolar surfaces, presented in Figure 29, exhibits a different surface phase diagram trend compared with those without H atoms, as presented in Figure 24 and Figure 26 [48,49]. Here, the surface phase diagrams are obtained assuming the H_2_ pressure (*p*_H2 _= 76 Torr (0.1 atm)) corresponds to H-rich conditions. The NH and NH_2_ H-terminated surfaces are primarily found over a wide range of temperatures and Al BEPs. Because of the wide growth chemical potential range, the calculated AlN polar surface phase diagrams are slightly different from the GaN surface.

The AlN(0001) surface phase diagram presented in Figure 29a demonstrates that the reconstructions with H atoms, such as 3Al–H and N_ad_–H + Al–H, emerge below 1520 K [48]. However, the surface without H atoms (N_ad_) can be formed above 1520 K even at H-rich conditions. This result suggests that there are several AlN(0001) surface reconstructions, and the growth processes may change drastically depending on temperature and Al pressure. Because the N dangling bonds in N_ad_ are chemically active compared with the N–H and Al–H bonds in 3Al–H and N_ad_–H + Al–H, the adsorption at high temperatures may be more efficient than at low temperatures. Furthermore, due to the presence of N_ad_ over a wide range of growth conditions at low H_2_ pressures, the growth rate at low H_2_ pressures is expected to be higher than at high H_2_ pressures. For the AlN$\left(000\bar{1}\right)$ surface, the N–H bonds (3N–H) are stabilized over a wide range of temperatures and Al pressures, as presented in Figure 29b, which suggests that the AlN$\left(000\bar{1}\right)$ surface growth processes are insensitive to the growth conditions.

The AlN$\left(1\bar{1}00\right)$ surface phase diagram presented in Figure 29c shows that the reconstruction with H atoms, 2N–H + 2Al–H, emerges below 1060 K, whereas the ideal surface without H atoms can be observed only above 1060 K [49]. The AlN$\left(11\bar{2}0\right)$ surface phase diagram presented in Figure 29d also shows that the 2N–H + 2Al–H appears in a very narrow temperature range (below 990 K), and the ideal surface forms over a wide range of growth temperatures and pressures. These results imply that during growth the AlN nonpolar surfaces always form the ideal surfaces, even under H-rich conditions. The growth processes on nonpolar orientations are expected to be unchanged by temperatures and Al pressures. In contrast to the other nitrides, such as GaN and InN, the AlN growth temperatures are too high to stabilize H atoms on nonpolar surfaces. The ideal surface appearance occurs upon growth due to H atom desorption. By comparing the AlN nonpolar surface phase diagrams with polar orientations, as presented in Figure 29a,b, it is expected that the AlN growth processes on nonpolar orientations are different from those on polar orientations.

### 4.3. Growth Kinetics

#### 4.3.1. Adatom Kinetics on Semipolar GaN Surfaces

It has also been reported that a GaN$\left(11\bar{2}2\right)$ surface with one Ga monolayer can be stabilized under Ga-rich conditions, and this surface is necessary to optimize the surface morphology in the plasma-assisted MBE [96]. Additionally, it is well known that $\left\{11\bar{2}2\right\}$ facets tend to appear on patterned GaN(0001) surfaces at low temperatures during conventional re-growth techniques [118,119,120]. These experimental results suggest that Ga-rich conditions are suitable for growth on GaN$\left(11\bar{2}2\right)$ surfaces. Although previous *ab initio* studies have elucidated some aspects of the growth kinetics, such as the adsorption and desorption behavior of Ga and N atoms on polar [75,121] and nonpolar [122] surfaces, semipolar GaN$\left(11\bar{2}2\right)$ surfaces have not been investigated theoretically.

**Figure 30:**
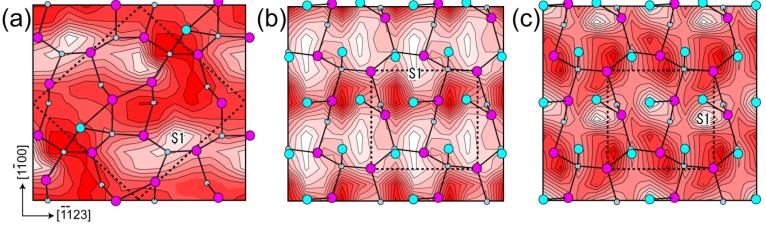
Contour potential energy surface (PES) plots for (**a**) an additional Ga atom on the GaN$\left(11\bar{2}2\right)$ surface with a Ga adatom and (**b**) Ga adatom and (**c**) N adatom on a GaN$\left(11\bar{2}2\right)$ surface with a Ga monolayer. Large and small circles represent Ga and N atoms, respectively. Each contour line in (**a**), (**b**), and (**c**) represents an energy step of 0.15, 0.05, and 0.15 eV, respectively. S1 represents stable adsorption sites. The dashed rectangles denote the surface unit cells.

For Ga and N adatoms on the surface with Ga dimers, the kinetics were determined by the potential energy surface (PES), which was calculated by fixing the adatom laterally at various positions and allowing relaxation along the vertical direction, as presented in Figure 16b. The PES calculations for the N adatom (not presented here) demonstrate that placing one N adatom close to the topmost N atom results in the formation of strong N-N bonds, and the N adatom desorbs together with the topmost N atom as an N_2_ molecule. The energy gain for N_2_ formation is 0.48 eV, suggesting that desorption occurs even at 0 K. Therefore, the adsorption of Ga adatoms to the outermost N atoms is necessary to form GaN layers on the surface. This is different from the adatom kinetics on the conventional GaN(0001) surface, in which the N-rich surface morphology can be kinetically stabilized [121]. For an additional Ga atom on the Ga dimer surface, calculations suggest that Ga atom desorption occurs easily under conventional growth conditions. Figure 30a illustrates the PES for the additional Ga atom on the surface. The most stable adsorption site is near the Ga-lattice site above the topmost N atom labeled S1 in Figure 30a and forms a Ga–N bond.

**Figure 31:**
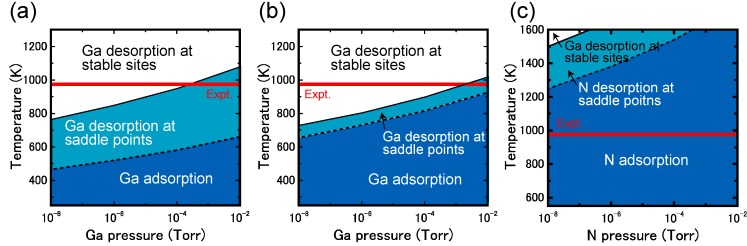
Calculated surface phase diagrams for Ga adsorption on (**a**) a GaN$\left(11\bar{2}2\right)$ surface with Ga adatom and (**b**) a GaN$\left(11\bar{2}2\right)$surface with a Ga monolayer and (**c**) N adsorption on a GaN$\left(11\bar{2}2\right)$ surface with a Ga monolayer as a function of temperature and pressure. The adsorption phase boundary at the stable sites (saddle points) is represented by solid (dashed) lines. The growth temperature in Refence [91] (~1000 K) is denoted by a horizontal line.

Figure 31a presents the surface phase diagram for the adsorption of an additional Ga atom on the Ga dimer surface. The adsorption temperature (solid line in Figure 31a) ranges from 770 to 1100 K depending on Ga pressure. The surface phase diagram suggests that most additional Ga atoms desorb under the MBE growth conditions (~1000 K) [96]. However, due to the small energy difference between *E*_ad_ and the gas-phase chemical potential, some Ga atoms will eventually adsorb. The Ga atom adsorption might not be entirely eliminated even during MBE growth. The PES presented in Figure 30a also shows an interesting result: the Ga migration behavior is different from on a GaN(0001) surface. The additional Ga atom migration barrier is 1.2 eV and is much higher than on the (0001) surface (~0.4 eV) [121], because only Ga-Ga bonds are formed at the saddle point instead of Ga–N bonds, which appear at stable and metastable positions. The high energy barrier results in a low adsorption energy at the saddle point (−1.76 eV), leading to notably low desorption temperatures, as shown by the dashed line in Figure 31a, compared to those at the stable sites. The desorption temperature at the saddle points is ~400 K lower than at the stable sites and suggests that even though Ga atoms eventually adsorb at the stable sites, they desorb during their surface migration.

The Ga and N adatom kinetics on the surface with a Ga monolayer, which corresponds to growth at low temperature or at high Ga pressures, are different from on the Ga dimer surface. The PES of a Ga adatom on the surface with a Ga monolayer, as presented in Figure 30b, exhibits an energetically smooth landscape in which the energy difference between the lowest and highest positions is within 0.89 eV. The stable adsorption site is located at the Ga-lattice site above the Ga monolayer labeled S1 in Figure 30b, and the most feasible migration pathway is along the 〈$\bar{1}\bar{1}23$〉 direction. The adsorption energy at S1, *E*_ad _= −2.73 eV, is comparable to the Ga dimer surface, but the migration energy barrier value (0.19 eV) is quite small. The Ga adatom surface phase diagram presented in Figure 31b indicates that Ga adatoms can adsorb at the stable sites, and some could reside on the surface during the migration during the MBE growth at high-Ga pressures. The low energy barrier on the Ga monolayer surface originates from the formation and dissociation of metallic Ga–Ga bonds near the saddle points.

Figure 30c presents the PES of an N adatom on the Ga monolayer surface. There is no adsorption site that forms N–N bonds, so desorption of an N_2_ molecule does not occur. The N adatom is incorporated at the N-lattice site between the Ga monolayer and the outermost Ga atom on the substrate, resulting in the formation of four Ga–N bonds that are labeled S1 in Figure 30c. This bond formation results in a small adsorption energy of −5.82 eV. At this adsorption energy, the surface phase diagram shows that for the N adatoms at stable sites, the desorption temperature exceeds 1500 K, as shown by the solid line in Figure 31c The N adatom migration energy barrier is 1.3 eV, leading to an adsorption energy below −4.60 eV at the saddle points. Using this value, as shown by the dashed line in Figure 31c, the desorption temperature ranges from 1200 to 1500 K depending on the N pressure. The N adatom can diffuse without desorption. Because these desorption temperatures are higher than during the MBE growth (~1000 K), we conclude that both Ga and N adatoms can be incorporated into the surface. Additionally, GaN layer formation proceeds under high-Ga pressure conditions and is consistent with experimental MBE results [96].

#### 4.3.2. Cubic GaN Growth Conditions

To eliminate polarization effects that are caused by the hexagonal (wurtzite) structure polarity, cubic (zinc blend structured) nitride layers could be used as an alternative. However, few studies have been published on cubic nitride growth compared with hexagonal nitride growth on GaN surfaces. Cubic GaN (*c*-GaN) is metastable, and thermally stable substrates at high temperatures are lacking. During the growth, experiments have shown that {111} facet formation causes hexagonal GaN (*h*-GaN) mixing, *i.e.*, in the regions grown toward the 〈111〉 directions, the stacking sequence of …*ABCABC*… easily collapses and changes into …*ABABAB*… stacking. [123] In addition, GaN(001)-(4 × 1) reconstruction to Ga-tetramers is stable under typical MBE growth conditions for *c*-GaN [124,125]. These results imply that two-dimensional growth without facet formation is important for the growth of single-phase *c*-GaN. To determine the *c*-GaN growth condition range, surface phase diagrams for the Ga and N atom adsorption process on a GaN (001) surface has been successfully applied [34,37].

**Figure 32:**
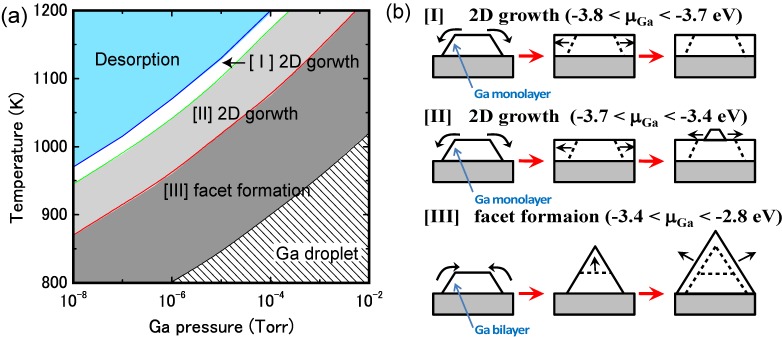
(**a**) Calculated phase diagrams for growth modes as a function of temperature a Ga BEP and (**b**) growth mode schematic representations with each corresponding to each temperature and Ga BEP region in the phase diagram.

If we assume that the {111} faceted surface appears, the stacking sequence of …*ABCABC*… easily changes to …*ABABAB*… stacking in the grown region along the 〈111〉 direction. Thus, the {111} faceted surface formation causes *h*-GaN mixing. Therefore, the stable conditions for the {111} faceted surface and for facet formation suppression should be clarified. Figure 32a displays the phase diagram for the growth conditions for *c*-GaN without {111} facet formation as a function of temperature and Ga BEP. This figure was obtained using the adsorption energy of Ga on a *c*-GaN(001)-(4 × 1) surface (−3.7 eV) [37] and the adsorption energy of artificial GaN(111) surfaces containing a Ga monolayer and bilayer, which seems to appear on the faceted surface. The adsorption energies on the Ga monolayer and bilayer are −3.8 and −3.4 eV, respectively) [74,126]. In regions [I] and [II] shown in Figure 24a, the gas-phase chemical potential of Ga, *μ*_Ga_, is lower than on the GaN(111) Ga-bilayer surface (−3.4 eV). Therefore, the Ga monolayer appears on the faceted surface, and the surface adsorption energy is −3.8 eV. For region [I], the *μ*_Ga_ is lower than the Ga adsorption energy on GaN(001)-(4 × 1) (−3.7 eV). In this region, impinging Ga adsorbs only on the (111) facetted surface. For region [II], the impinging Ga adsorbs on the facetted surface and then adsorbs on the (001) surface because *E*_ad-Ga(111)_ < *E*_ad-Ga(001)_ < μ_Ga_, where *E*_ad-Ga(hkl)_ is the Ga adsorption energy on the (*hkl*) surface. For region [III], the *μ*_Ga_ is higher than the Ga adsorption energy on the Ga-bilayer surface (−3.4 eV). Therefore, the Ga bilayer surface appears on the facetted surface for these growth conditions. In this region, impinging Ga atoms adsorb on the (001) surface and then adsorb on the facetted surface because *E*_ad-Ga(001)_ < *E*_ad-Ga(111)_ < μ_Ga_. These results suggest that two-dimensional growth occurs when the growth conditions are within regions [I] and [II]. However, facet formation occurs when the growth condition is within region [III], as presented in Figure 32b. We thus concluded that by choosing appropriate growth conditions, the growth formation and *h*-GaN mixing can be controlled.

#### 4.3.3. Adatom Kinetics on Polar AlN Surfaces

The AlN growth condition optimization could also be achieved with an understanding of the growth mechanisms. However, the AlN surface atomic-scale growth processes, such as adatom kinetics during epitaxial growth, still remain unclear. Theoretical studies on the properties and growth of AlN are sparse and have focused on the atomic and electronic structure [48,49,105,113,116]. Recent *in situ* reflectance data of AlN films grown with MOVPE have reported that the growth rate on AlN(0001) surfaces significantly depends on the carrier gas species [127]. In addition, they report that growth under N-rich conditions is much faster than under H-rich conditions. This experimental finding can be interpreted using the supersaturation of Al, depending on the partial pressure of H_2_ [128], but the effects of the AlN(0001) surface growth processes during MOVPE growth have not been examined. Detailed AlN surface-adatom kinetic studies would provide a deeper understanding of the growth processes. Indeed, calculations for the adsorption and diffusion behaviors of Al and N atoms on technologically relevant AlN(0001) surfaces during MOVPE growth have revealed that the surface reconstruction crucially affects the adatom kinetics [50].

**Figure 33:**
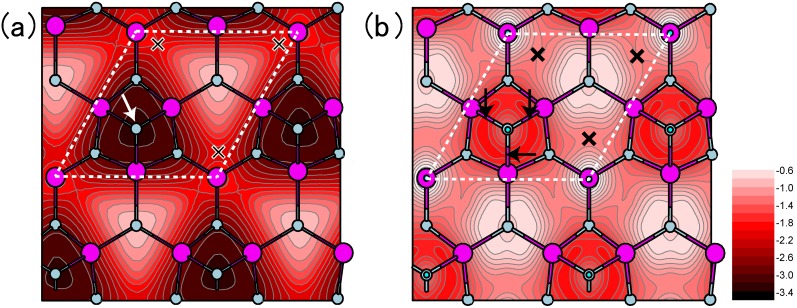
Contour PES plots for an Al adatom on reconstructed AlN(0001) surfaces with (**a**) an N adatom and (**b**) H atoms. Large, small, and tiny circles represent Al, N, and H atoms, respectively. Each contour line in (**a**) and (**b**) represents an energy step of 0.1 and 0.2 eV, respectively. The dashed rectangles denote the surface unit cells. Arrows and crosses in the unit cell represent minima and PES saddle points, respectively.

Based on the calculated surface structures presented in Figure 29, the kinetics of Al and N adatoms on the AlN(0001) surface have been examined. The PES calculations for an N adatom on the surface with another N adatom and with H atoms show that the most stable adsorption site for the additional N atom is located near the pre-adsorbed N atom. However, the adsorption energies (~2.29 eV) indicate that the N atom desorption occurs even at 0 K. This finding thus suggests that the adsorption of Al adatoms that attach to the outermost N atom is necessary to form AlN layers on AlN(0001) surfaces. These results are contradictory to the adatom kinetics on a GaN(0001) surface, in which N-rich surface morphology can be kinetically stabilized [121]. The PES calculations for an Al adatom on the surface suggest that the Al atom adsorption behavior significantly depends on the reconstruction. Figure 33a,b presents the PES of an additional Al atom on surfaces with an N adatom and H atom, respectively. As shown in Figure 25a, the most stable adsorption site on the surface under low H_2_ pressure conditions is located above the N adatom, and a strong Al–N bond with a bond length of 1.81 Å is formed, which is similar to the bond length in bulk AlN (1.91 Å). The Al–N bond formation results in an adsorption energy of *E*_ad _= −3.25 eV, which is much lower than the adsorption energy at high H_2_ pressure conditions. To move adjacent stable adsorption sites, there is a transition state for diffusion. The Al adatom positions in the transition state are located close to the topmost Al atom, which does not have an Al–N bond with the N adatom. The energy barrier for diffusion is 0.81 eV. This transition state corresponds to the dissociation of an Al–N bond between the N adatom and the topmost Al atom.

However, the most stable surface adsorption sites under high H_2_ pressure conditions are located between topmost Al–N bonds, as shown in Figure 33b. An Al–Al bond (bond length 2.66 Å) is formed between the Al adatom and topmost surface Al atom. The adsorption energy is *E*_ad_ = −1.89 eV, corresponding to the energy required to form an Al–Al bond. The energetically lowest transition sites for diffusion are located close to the hexagonal sites without an N adatom, and the corresponding energy barrier *E*_diff_ is 0.75 eV. The physical origin of the energy barrier is attributed to the formation of a weak Al–Al bond. This bond is stretched by 24% from the original Al–Al bond, indicating a significantly reduced bond strength. Despite the small energy barrier difference, which depends on the growth conditions, the physical origin of the energy barrier is quite different.

**Figure 34:**
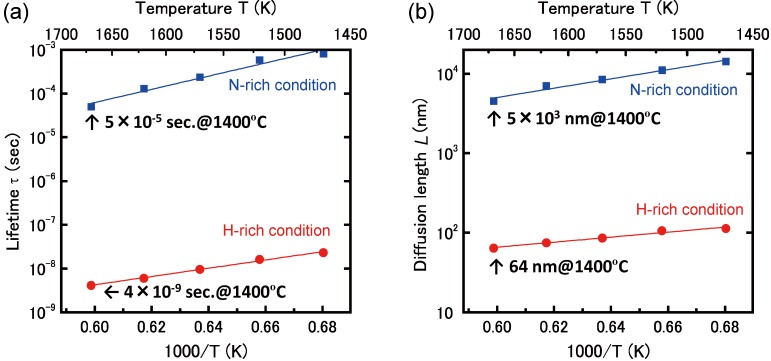
Calculated (**a**) life time, *τ*, and (**b**) diffusion length, *L*_diff_, of an Al adatom on a AlN(0001) surface as a function of reciprocal temperature at an Al pressure of 1 × 10^−3^ Torr as obtained by kinetic Monte Carlo simulations.

A more qualitative adatom kinetic analysis can be performed using the calculated adsorption energies and diffusion barriers. This analysis was carried out by estimating the diffusion length, *L*_diff_, expressed as $L_{diff}=\sqrt{2D\tau }$, where *D* is the diffusion coefficient and *τ* is the life time of the Al adatom between the adsorption and desorption events. The difference in *E*_diff_, which is within 0.06 eV depending on the reconstruction, does not contribute to the difference in diffusion length because *D* is proportional to $\text{exp}\left(-E_{diff}/k_{B}T\right)$. On the contrary, the desorption probability defined by *τ*^−1^ is proportional to $\text{exp}\left(-E_{de}/k_{B}T\right)$, where $E_{\text{de}}=-E_{\text{ad}}$ is the desorption energy. The desorption energy of an Al adatom, depending on the growth conditions, thus affects the diffusion length. Figure 34a,b presents the estimated lifetime, *τ*, and diffusion length, *L*_diff_, of an Al adatom under low and high H_2 _pressure conditions (at an Al pressure of 1 × 10^−3^ Torr) using the kinetic Monte Carlo simulations described in Section 4.2. The estimated *τ* and *L*_diff_ for low H_2_ pressure conditions are four and two orders of magnitude larger than for high H_2 _pressure conditions, respectively. This indicates that growth under N-rich conditions is much faster than under H-rich conditions. Although the adsorption processes for a monolayer AlN film should be verified to obtain the growth rate more quantitatively, this conclusion is qualitatively consistent with recent *in situ* reflectance data of AlN films grown with MOPVE [127].

### 4.4. Impurity Incorporation

#### 4.4.1. Mg Incorporation on GaN Surfaces

The control of the charge-carrier concentration through doping is a key issue for many nitride semiconductor applications. The discovery of p-type conductivity in GaN surfaces that are Mg doped has led to the widespread development of GaN-based optoelectronic devices [129,130]. Recently, Mg incorporation has been found to be more efficient on semipolar GaN$\left(1\bar{1}01\right)$ surfaces than on polar GaN(0001) surfaces. Secondary ion-microprobe mass spectrometry (SIMS) measurements have reported that Mg concentrations on the GaN$\left(1\bar{1}01\right)$ surface were are higher than on the polar (0001) surface [131,132]. However, the ideal cleavage GaN$\left(1\bar{1}01\right)$ surface is an N-terminated surface similar to the ideal GaN$\left(000\bar{1}\right)$ surface because the Mg doping efficiency is rather poor [133]. Therefore, the origins of high Mg concentrations on the N-terminated GaN$\left(1\bar{1}01\right)$ surface cannot be explained by the GaN$\left(000\bar{1}\right)$ surface. To clarify the origin of high Mg concentrations on the semipolar $\left(1\bar{1}01\right)$ orientation, theoretical studies to discern the Mg-incorporation behavior are necessary. Theoretical studies on the stability of Mg on GaN surfaces have been performed to address many issues raised by experimental results. To explain the narrow window for GaN smooth growth due to Mg on the GaN(0001) surface [134], the relative stabilities of possible Mg-rich reconstructions have been determined with respect to the clean surface. Surface structures comprised of a 1/2 to 3/4 monolayer of Mg substituted for Ga have been proposed under very Mg-rich conditions [51]. The energetics of Mg adsorption and incorporation on the GaN(0001) and $\left(000\bar{1}\right)$ surfaces under a wide variety of conditions has been determined using *ab initio* calculations [52]. Mg incorporation proceeds on the Ga-polar surface, but high Mg coverage tends to locally change the polarity from Ga to N polar. A thermodynamic approach that includes chemical potentials that are appropriate for realistic growth conditions has revealed that hydrogen stabilizes Mg-rich surface reconstructions for both GaN(0001) and $\left(1\bar{1}00\right)$ surfaces [135]. In addition, the stability of the Mg-incorporated GaN$\left(1\bar{1}0\bar{1}\right)$ surface has been examined [40] to explain high hole concentrations in the Mg-doped semipolar GaN$\left(1\bar{1}0\bar{1}\right)$ surface [136].

**Figure 35:**
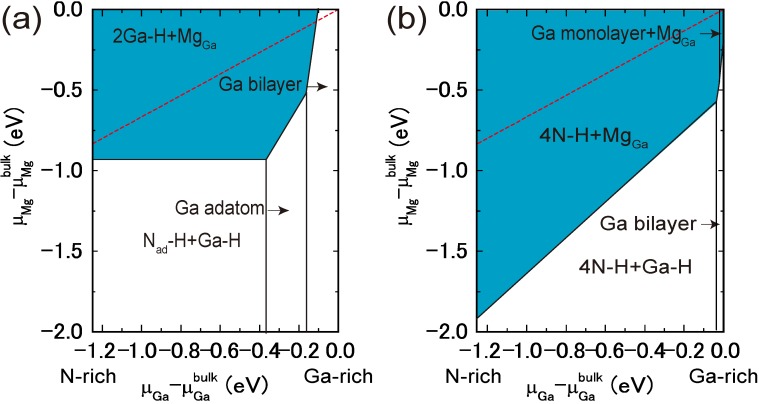
Stable Mg-incorporated structures on (**a**) GaN(0001) and (**b**) GaN$\left(1\bar{1}01\right)$ surfaces as a function of Ga chemical potential, *μ*_Ga_, and Mg chemical potential, *μ*_Mg_, under high H_2_ pressure conditions (*μ*_H_ = (1/2)*E*_H2 _− 1.05 eV, where *E*_H2_ is total energy of a H_2_ molecule). The stable regions of Mg-incorporated surfaces are emphasized by the shaded areas.

Figure 35 depicts a diagram of stable structures on GaN$\left(1\bar{1}01\right)$ and GaN(0001) surfaces including Mg atoms as a function of μ_Ga_ and μ_Mg_ at high H_2_ pressure obtained using Equation (1) [44]. The boundary lines separating different regions correspond to chemical potentials for two structures that have the same formation energy. Here, a single Mg atom in the unit cell is assumed because the Mg partial pressure during doping should be considerably lower than the Ga partial pressure, *i.e.*, the Mg chemical potential is expected to vary less than for the Mg-rich limit for the Mg_3_N_2_ precipitation. These diagrams demonstrate that Mg atoms can be incorporated. However, remarkable orientation dependence exists for the stable Mg-incorporated surface regions. As presented in Figure 35b for the GaN$\left(1\bar{1}01\right)$ surface, the H-terminated surface with a substituted Mg (4N–H + Mg_Ga_) is stabilized over a wide Ga chemical potential range. Although the stable Mg-incorporated structures at high H_2_ pressures are different from those at low H_2_ pressures, the stable Mg-incorporated surface regions are similar to those at low H_2_ pressures between the phase space at high H_2_ pressures that have a range of chemical potentials [44]. In contrast, for the GaN(0001) surface, the Mg-incorporated surface stable region is drastically reduced by the presence of hydrogen. For *μ*_Mg_ ≤ −0.91 eV, the H-terminated surface with a N adatom (N_ad_–H + Ga–H) shown in Figure 35a is stabilized over a wide Ga chemical potential range. Strong Ga–N and N–H bonds are formed in N_ad_–H + Ga–H, which simultaneously satisfies the EC rule [79]. For the N-rich conditions, the stabilization of N_ad_–H + Ga–H leads to a narrow *μ*_Mg_ range where the Mg-incorporated surface is stabilized.

**Figure 36:**
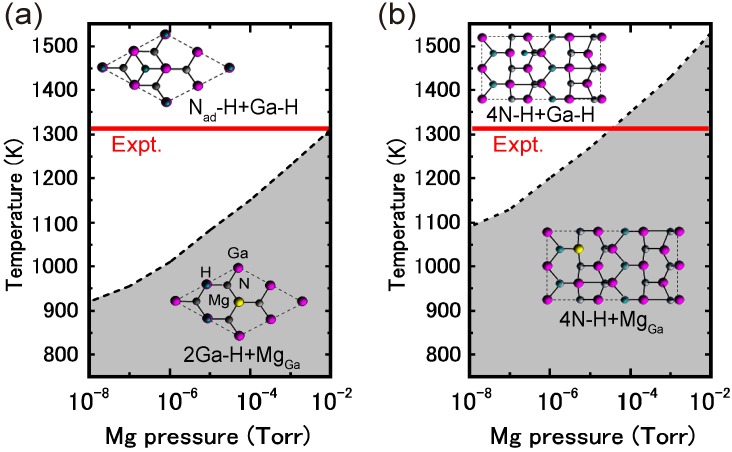
Calculated temperatures for the phase transition between Mg-incorporated and Mg-free surfaces at the N-rich limit (*μ*_Ga_ = −1.24 eV) as a function of Mg BEP on the (**a**) GaN(0001) and (**b**) GaN$\left(1\bar{1}01\right)$ surfaces. The Mg-incorporated surfaces are stabilized in the shaded regions. The GaN MOVPE growth temperature was provided by References [131,132] and is denoted by red lines.

To discuss the orientation dependence for the Mg-incorporated surface stabilities, the phase transition between the Mg-incorporated and Mg-free surfaces in the N-rich limit has been estimated using surface phase diagrams [44]. Figure 36 presents the phase transition temperatures on the GaN$\left(1\bar{1}01\right)$ and GaN(0001) surfaces at *p*_H2_ = 76 Torr. For the GaN(0001) surface shown in Figure 36a, the transition temperatures at *p*_H2_ = 76 Torr ranges from 930 to 1310 K. In contrast, as presented in Figure 36b, the transition temperatures at *p*_H2_ = 76 Torr (1090–1530 K) are remarkably higher than those on the GaN(0001) surface. The orientation dependence on the transition temperatures originates from the difference in the boundary line between the surfaces with and without Mg, as presented in Figure 35. The lower phase transition temperatures for GaN(0001) suggest that during MOPVE growth at approximately 1300 K, the incorporation of Mg atoms on the semipolar $\left(1\bar{1}01\right)$ orientation is more efficient than on the polar (0001) orientation. Although the kinetics, such as the adsorption and desorption behavior of Mg, for the growth processes on a large unit cell should be verified, the efficient Mg incorporation results in high Mg concentrations on the GaN$\left(1\bar{1}01\right)$ surface, which is qualitatively consistent with experimental SIMS results [131,132].

#### 4.4.2. C Incorporation on GaN Surfaces

In addition to Mg doping, carbon also acts as a p-type dopant if it is incorporated on the nitrogen lattice site [137]. However, successful p-type doping by carbon has never been reported on the conventional GaN(0001) surface. Previous experiments have reported that carbon doping on this orientation results in the formation of deep grooves [138]. However, p-type conductivity has been successfully obtained by carbon doping on semipolar GaN$\left(1\bar{1}01\right)$ surfaces [139,140]. These experimental findings suggest that the difference in doping behavior between (0001) and $\left(1\bar{1}01\right)$ orientations is attributed to the surface polarity because the ideal GaN(0001) and $\left(1\bar{1}01\right)$ surfaces are terminated by Ga and N faces, respectively. This difference results in a difference in carbon substitution efficiency on the nitrogen lattice sites. However, at present little is known concerning the stability and structure of carbon incorporated surfaces.

**Figure 37:**
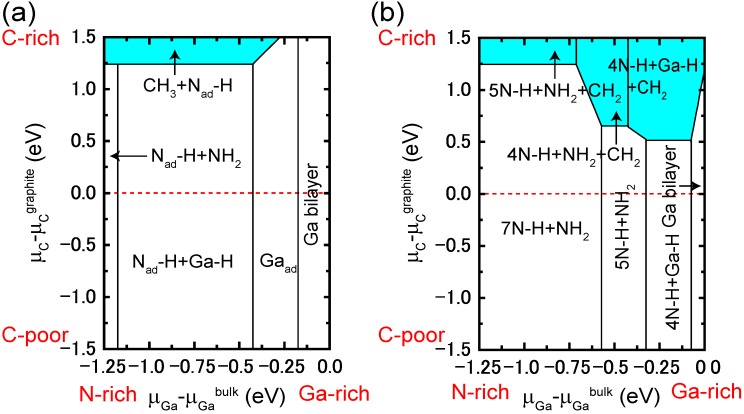
Stable structures of carbon incorporated (**a**) GaN(0001) and (**b**) GaN$\left(1\bar{1}01\right)$ surfaces as a function of Ga chemical potential, *μ*_Ga_, and C chemical potential, *μ*_C_, for high H_2_ pressure conditions (*μ*_H_ = (1/2)*E*_H2_ − 1.05 eV, where *E*_H2_ is total energy of a H_2_ molecule). The stable carbon incorporated surface regions are shaded.

Figure 37 depicts the diagrams of stable structures on GaN(0001) and $\left(1\bar{1}01\right)$ surfaces including carbon as a function of *μ*_Ga_ and *μ*_C_ at high H_2_ pressure obtained using Equation (1) [46]. These diagrams demonstrate that carbon-free surfaces, such as 7N–H + NH_2_ (the surface with N–H bonds and NH_2_), are stabilized over a wide range of *μ*_Ga_ and *μ*_C_. However, a carbon atom can be incorporated under C-rich conditions for both GaN(0001) and $\left(1\bar{1}01\right)$ surfaces. Furthermore, there is an orientation dependence in the stabilization of the carbon-incorporated structure. For the GaN(0001) surface, as presented in Figure 37a, the surface with CH_3_ and H-terminated N adatoms (CH_3_ + N_ad_–H) is stabilized for *μ*_C_ − $\mu_{C}^{\text{graphite}}$ ≥ −1.24 eV under N-rich and moderate Ga-rich conditions, where $\mu_{C}^{graphite}$ is the chemical potential of graphite. This structure has CH_3_ replacing NH_2_ in the hydrogen-terminated surface containing NH_2_ and N adatoms (N_ad_–H + NH2), which suggests that under C-rich conditions C atoms can be preferentially adsorbed on the N lattice site of the GaN(0001) surface. In contrast, the chemical potential range for the carbon incorporated GaN$\left(1\bar{1}01\right)$ surface stabilization, as presented in Figure 37b, is larger than for the GaN(0001) surface. The surface with CH_2_ at the Ga lattice site (5N–H + NH_2_ + CH_2_) is *μ*_C_ − $\mu_{C}^{\text{graphite}}$ ≥ −1.24 eV under N-rich conditions, and those with CH_2_ at the N lattice site (4N–H+NH_2_+CH_2_ and 4N–H+Ga–H+CH_2_) are stable for *μ*_C_ − $\mu_{C}^{\text{graphite}}$ ≥ −0.5 eV under Ga-rich conditions. Therefore, p-type conductivity on the GaN$\left(1\bar{1}01\right)$; surface can be achieved via the formation of 5N–H + NH_2_ + CH_2_ (4N–H + NH_2_ + CH_2_ and 4N–H + Ga–H + CH_2_) under N-rich (Ga-rich) conditions. The orientation dependence in the stability of carbon incorporated surfaces is due to the formation of Ga–C (N–C) bonds. The energy profits caused by two Ga–C and N–C bonds (5.8 and 5.0 eV, respectively) on the carbon incorporated GaN$\left(1\bar{1}01\right)$ surface are larger than for a single Ga–C bond (3.1 eV) on the GaN(0001) surface. All the carbon incorporated structures are thermodynamically unstable against the formation of graphite. However, C incorporation that is higher than in graphite would occur during the growth due to larger amounts of C in C_2_H_4_ and CCl_4_, which are used as the source gases [139,140]. It is likely that the high reaction energy of graphite from these source gases prevents graphite formation during GaN growth.

**Figure 38:**
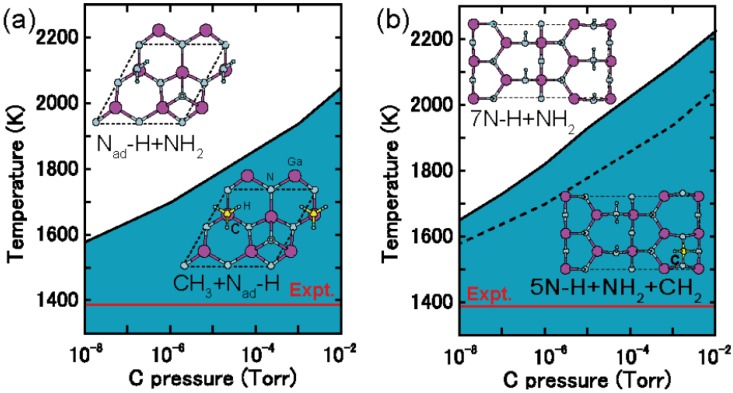
Calculated surface phase diagrams for C-incorporated (**a**) GaN(0001) and (**b**) $\left(1\bar{1}01\right)$ GaN surfaces at the N-rich limit (*μ*_Ga_ = −1.24 eV) as a function of temperatures and C BEP. The carbon incorporated surfaces are stabilized in the shaded regions. Schematic views of surface structures are also presented. The growth temperature from [139,140] are shown by red lines. For comparison, the dashed line in (**b**) denotes the phase boundary between carbon free and incorporated surfaces on the GaN(0001) surface.

Finally, Figure 38 displays the calculated surface phase diagrams for the desorption of carbon atoms from GaN(0001) and $\left(1\bar{1}01\right)$ surfaces as a function of temperature and C BEP [46]. These surface phase diagrams suggest that for both GaN(0001) and $\left(1\bar{1}01\right)$ surfaces the incorporation of carbon during the growth is efficient at low temperatures. For the GaN(0001) surface, as presented in Figure 38a, the temperatures range from 1550–2050 K depending on the C pressure for the desorption of C on CH_3_ + N_ad_–H. In contrast, the desorption temperatures of C on 5N–H + NH_2_ + CH_2_ on the GaN$\left(1\bar{1}01\right)$ surface range from 1660 to 2220 K, as presented in Figure 38b. These temperatures are higher than those on the GaN(0001) surface presented in Figure 38a. This temperature difference leads to the orientation dependence on the stability under growth conditions. Considering the kinetics, such as surface migration, more carbon atoms on the GaN(0001) surface compared to the GaN$\left(1\bar{1}01\right)$ surface could desorb from the surface during the growth processes. We thus expect that the C atom concentration on the GaN$\left(1\bar{1}01\right)$ surface is larger than on the GaN(0001) surface. The high carbon concentrations result in p-type doping only on the GaN$\left(1\bar{1}01\right)$ surface. Additionally, the most stable adsorption site under N-rich conditions in 5N–H + NH_2_ + CH_2_ is located at the Ga lattice site. If this C atom is stably located at the Ga lattice site during the growth processes, p-type conductivity cannot be explained by this structure. To find the percentage of carbon that is ionized and releases holes on the GaN$\left(1\bar{1}01\right)$ surface, detailed studies of the carbon atom adsorption and desorption behaviors during the growth processes should be performed.

## 5. Conclusions

In this review, we explained the feasibility and versatility of our *ab initio*-based approach that incorporates the gas-phase free energy. The calculated surface phase diagrams of GaAs(001) Ga-rich and As-rich surfaces agreed well with the experimental results. We also compared the theoretical and experimental Ga diffusion lengths on the GaAs(001)-(2 × 4)β2 surface, which showed good agreement. This suggests that the *ab initio*-based approach is a powerful tool for investigating the surface stability and growth kinetics in the VPE of compound semiconductors.

This *ab initio*-based approach was applied to various phenomena on nitride semiconductor surfaces. The reconstruction, adsorption and incorporation on various nitride surfaces were systematically investigated. The calculated results for surface reconstructions with polar, nonpolar, and semipolar orientations suggest that the reconstructions on nitride surfaces with adlayers appear on the polar and semipolar surfaces. However, low cation coverage is favorable on the nonpolar surfaces. The hydrogen-terminated surfaces with N–H and NH_2_ were primarily found on nitride surfaces. These hydrogen terminated surfaces were stabilized to satisfy the EC rule [79]. There were several hydrogen-adsorbed surface structures with polar and nonpolar orientations that formed N–H and NH_2_, depending on the temperature and BEP. In contrast, the most stable hydrogen-adsorbed structures on the semipolar surfaces did not vary over the wide range of temperature and BEP. These results imply that the hydrogen-adsorbed structures will emerge depending on the growth conditions of the polar and nonpolar orientations and regardless of the semipolar-orientation growth conditions during the MOVPE growth.

The Ga and N atom adsorption and desorption behavior and migration on semipolar GaN$\left(11\bar{2}2\right)$ surfaces were also investigated. The migration energy barrier for an additional Ga atom on the surface with Ga dimers (1.2 eV) is much higher than on the conventional GaN(0001) surface (0.4 eV), which leads to the desorption of both Ga and N atoms under conventional growth conditions. In contrast, on the surface with a Ga monolayer, both Ga and N atoms are adsorbed and migrate without desorption. Either low temperature or high Ga pressure is required to grow GaN on the $\left(11\bar{2}2\right)$ orientation. The growth conditions of *c*-GaN with a two-dimensional growth mode were determined based on the adsorption of Ga on GaN(001)-(4 × 1) and GaN(111) surfaces. The growth conditions for stabilizing the {111} faceted surfaces were clarified, and *c*-GaN can be grown using the two-dimensional growth mode without {111} facet formation when the proper growth conditions are chosen, suggesting the possibility of *c*-GaN growth without *h*-GaN mixing. The adsorption of an Al adatom on a AlN(0001) surface strongly depends on the surface reconstruction, whereas its diffusion is not affected by the reconstruction. The adsorption of an Al adatom on a AlN(0001) surface under N-rich conditions is much easier than under H-rich conditions, suggesting that AlN growth during MOVPE dominates under N-rich rather than H-rich conditions.

Additionally, we found that dopant (Mg and C) incorporation behavior on the GaN(0001) and GaN$\left(1\bar{1}01\right)$ surfaces is strongly affected by hydrogen adsorption. Hydrogen adsorption enhances the dopant stability on a semipolar GaN$\left(1\bar{1}01\right)$ surface. The stabilization conditions were similar for both the H-terminated surfaces with Mg at the Ga lattice site at high H_2_ pressures and for those at low pressures on the GaN$\left(1\bar{1}01\right)$ surface. In contrast, the stable conditions are rather severe for Mg-incorporated surfaces under high H_2_ pressures for the GaN(0001) surface due to the stabilization of the Mg-free surface with H-terminated N adatom, which results in orientation dependence. This result provides a possible explanation for the experimental data in which GaN exhibits rather high Mg concentrations when GaN is fabricated on the semipolar $\left(1\bar{1}01\right)$ orientation by the MOVPE growth method. The stable conditions for C-incorporated surfaces were found to be more pronounced for the GaN(0001) surface compared with the GaN$\left(1\bar{1}01\right)$ surface. The orientation dependence of the C-incorporated surface stability provides a possible explanation for p-type doping on the semipolar GaN$\left(1\bar{1}01\right)$ surface. Although these calculated results should be compared with additional future experiments, the *ab initio*-based approach, which takes the growth parameters into account, is feasible not only for investigating surface structures but also for clarifying adsorption and incorporation processes on semiconductor surfaces.
